# Combined transcriptome and proteome analysis reveal the key physiological processes in seed germination stimulated by decreased salinity in the seagrass *Zostera marina* L.

**DOI:** 10.1186/s12870-023-04616-x

**Published:** 2023-11-30

**Authors:** Yu Zhang, Shidong Yue, Mingjie Liu, Xinhua Wang, Shaochun Xu, Xiaomei Zhang, Yi Zhou

**Affiliations:** 1 CAS Key Laboratory of Marine Ecology and Environmental Sciences, Institute of Oceanology, Chinese Academy of Sciences, Qingdao, 266071 China; 2 Laboratory for Marine Ecology and Environmental Science, Qingdao National Laboratory for Marine Science and Technology, Qingdao, 266237 China; 3 Center for Ocean Mega-Science, Chinese Academy of Sciences, Qingdao, 266071 China; 4 CAS Engineering Laboratory for Marine Ranching, Institute of Oceanology, Chinese Academy of Sciences, Qingdao, 266071 China; 5 University of Chinese Academy of Sciences, Beijing, 100049 China; 6 Shandong Province Key Laboratory of Experimental Marine Biology, Qingdao, 266071 China

**Keywords:** Seagrass, *Zostera marina* L., Seed germination, Low-salinity stimulation, Transcriptome and proteome

## Abstract

**Background:**

*Zostera marina* L., or eelgrass, is the most widespread seagrass species throughout the temperate northern hemisphere. Unlike the dry seeds of terrestrial plants, eelgrass seeds must survive in water, and salinity is the key factor influencing eelgrass seed germination. In the present study, transcriptome and proteome analysis were combined to investigate the mechanisms via which eelgrass seed germination was stimulated by low salinity, in addition to the dynamics of key metabolic pathways under germination.

**Results:**

According to the results, low salinity stimulated the activation of Ca^2+^ signaling and phosphatidylinositol signaling, and further initiated various germination-related physiological processes through the MAPK transduction cascade. Starch, lipids, and storage proteins were mobilized actively to provide the energy and material basis for germination; abscisic acid synthesis and signal transduction were inhibited whereas gibberellin synthesis and signal transduction were activated, weakening seed dormancy and preparing for germination; cell wall weakening and remodeling processes were activated to provide protection for cotyledon protrusion; in addition, multiple antioxidant systems were activated to alleviate oxidative stress generated during the germination process; ERF transcription factor has the highest number in both stages suggested an active role in eelgrass seed germination.

**Conclusion:**

In summary, for the first time, the present study investigated the mechanisms by which eelgrass seed germination was stimulated by low salinity and analyzed the transcriptomic and proteomic features during eelgrass seed germination comprehensively. The results of the present study enhanced our understanding of seagrass seed germination, especially the molecular ecology of seagrass seeds.

**Supplementary Information:**

The online version contains supplementary material available at 10.1186/s12870-023-04616-x.

## Background

Seagrasses are marine angiosperms that form large shallow meadows with high ecological value [1–5]. However, seagrass species diversity and coverage have declined dramatically globally due to both natural and anthropogenic disturbance, which have caused the decline of the critical ecosystem globally [6, 7]. To halt and reverse such losses, attempts are already underway to restore seagrass meadows, various strategies have been adopted [6, 8–12], and seeding has become a widely used restoration method [9, 13, 14].

*Zostera marina* L., or eelgrass, is the most widespread seagrass species throughout the temperate northern hemisphere of the Pacific and Atlantic [15, 16], and is also one of the most threatened seagrass species. In recent years, much efforts of restoration based on eelgrass seeds have been carried out worldwide [6, 13, 14]. Current studies on eelgrass seed germination have focused on seed ecology, mainly on the effects of temperature, salinity, light, sediment type, burial depth, oxygen potential, and other factors, on seed germination [17]. Unlike the dry seeds of terrestrial plants, eelgrass seeds must survive in water [18, 19], and salinity is the key factor influencing eelgrass seed germination, low salinity promoted seed germination, while high salinity inhibited it [19, 20]. Nowadays, excessive rainfall caused by climatic anomalies may decrease the seawater salinity in seagrass beds in coastal zone [21, 22], which will affect seagrass seed germination. Seed germination is regulated both by the external environment and internal molecular mechanisms. Nonetheless, few studies have explored the molecular dynamics behind seagrass seed germination.

Seed germination is the beginning of the second cycle of a plant’s life [23]. Germination is the physiological process by which a seed begins to absorb water until the radicle emerges [24]. During terrestrial monocotyledon seed germination, the cotyledon and radicle are covered by a coleoptile and coleorhiza, respectively, and the coleorhiza and radicle grow out of the seed in sequence, following which the coleoptile is pushed upward to the surface [25–27]. Although eelgrass is a kind of monocotyledon, the physiological process of eelgrass seed germination is slightly different compared to that of terrestrial monocotyledon seeds germination: eelgrass seeds do not produce radicles; the hypocotyl develops into a swollen basal part (the original embryonic mass) and an elongated axial part formed by cell divisions at the base of the cotyledon and plumule; the first leaflike structure is the cotyledon [28, 29]. Numerous complex physiological processes are involved in seed germination, including plant hormone metabolism and signal transduction, nutrient and energy metabolism, cell wall remodeling and modification, and DNA damage and repair [30, 31]. The exploration of the mechanisms controlling seed germination has been facilitated greatly by significant advances in genomics research. Gene expression analyses at the RNA and protein levels have been used to reveal seed dormancy and germination characteristics [31–33]. In comparison, transcriptomic analysis is more widely applied; however, the seed germination process involves the reactivation of a series of physiological and biochemical reactions, which are catalyzed or mediated by different proteins, so that it would be valuable to integrate analyses of protein fractions during seed germination.

To investigate the molecular dynamics of seagrass seed germination (genetic process) and mechanisms by which low salinity stimulated eelgrass seed germination, the present study integrated the transcriptomic and proteomic profiles of eelgrass seed germination under low salinity stimulation, and analyzed the key metabolic pathways during germination in detail. The results of the present study enhanced our understanding of seagrass seed biology and could facilitate future seagrass improvement and conservation efforts.

## Results

### Basic transcriptomic and proteomic data

**Fig. 1:**
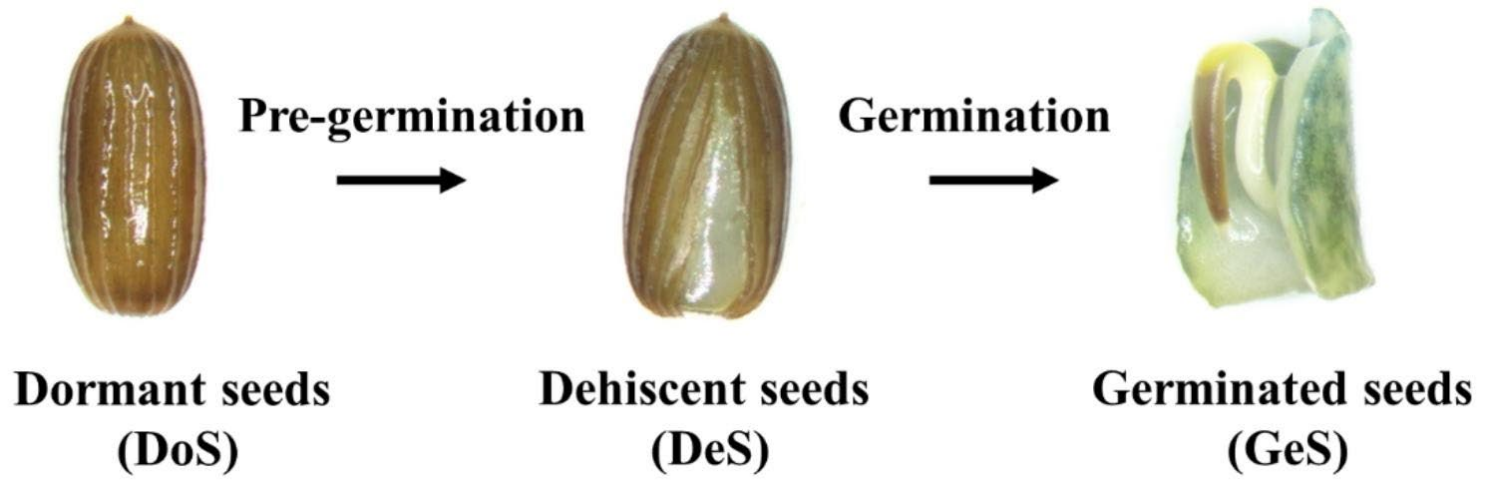
Three stages of eelgrass seed during germination

**Fig. 2:**
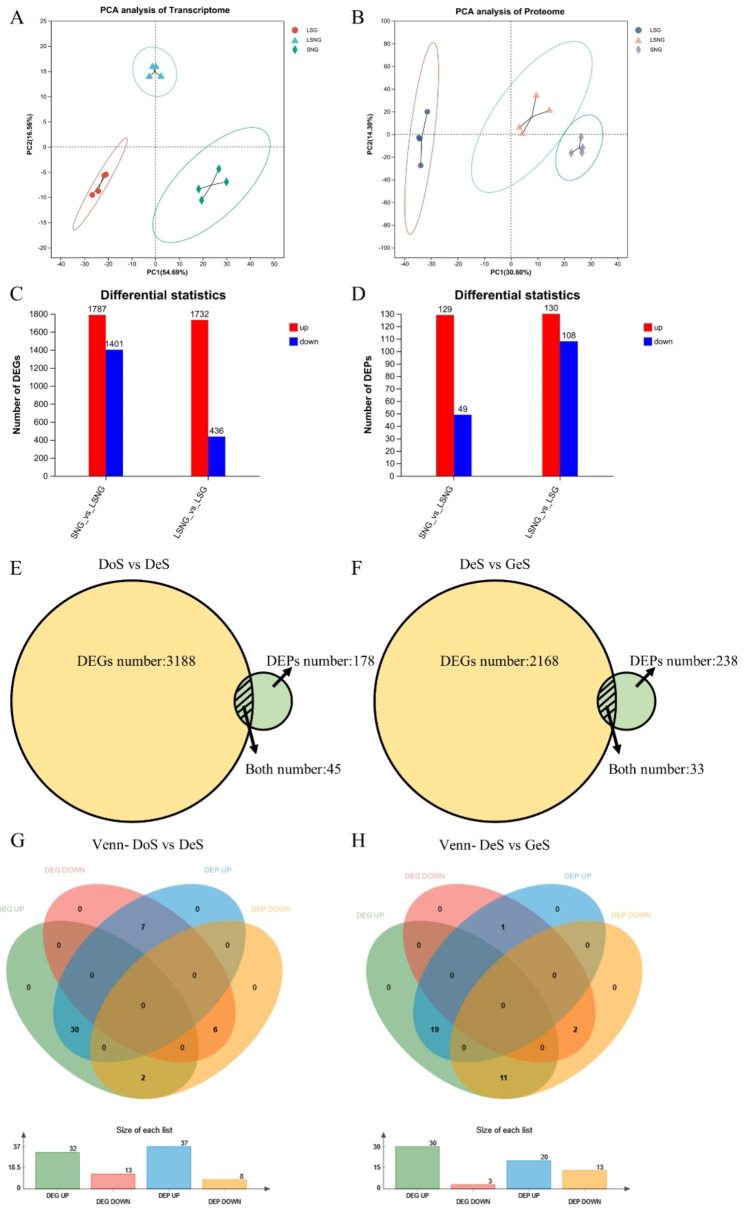
Basic analysis of transcriptomic and proteomic data. (**a, b**) Principal component analysis (PCA) of transcriptome and metabolome. (**c, d**) Differentially expressed genes (DEGs) and differentially expressed proteins (DEPs) statistics. (**e, f**) Association diagram of DEGs and DEPs. (**g, h**) Venn analysis of DEGs and DEPs. DoS, Dormant seeds; DeS, Dehiscent seeds; GeS, Germinated seeds

**Fig. 3:**
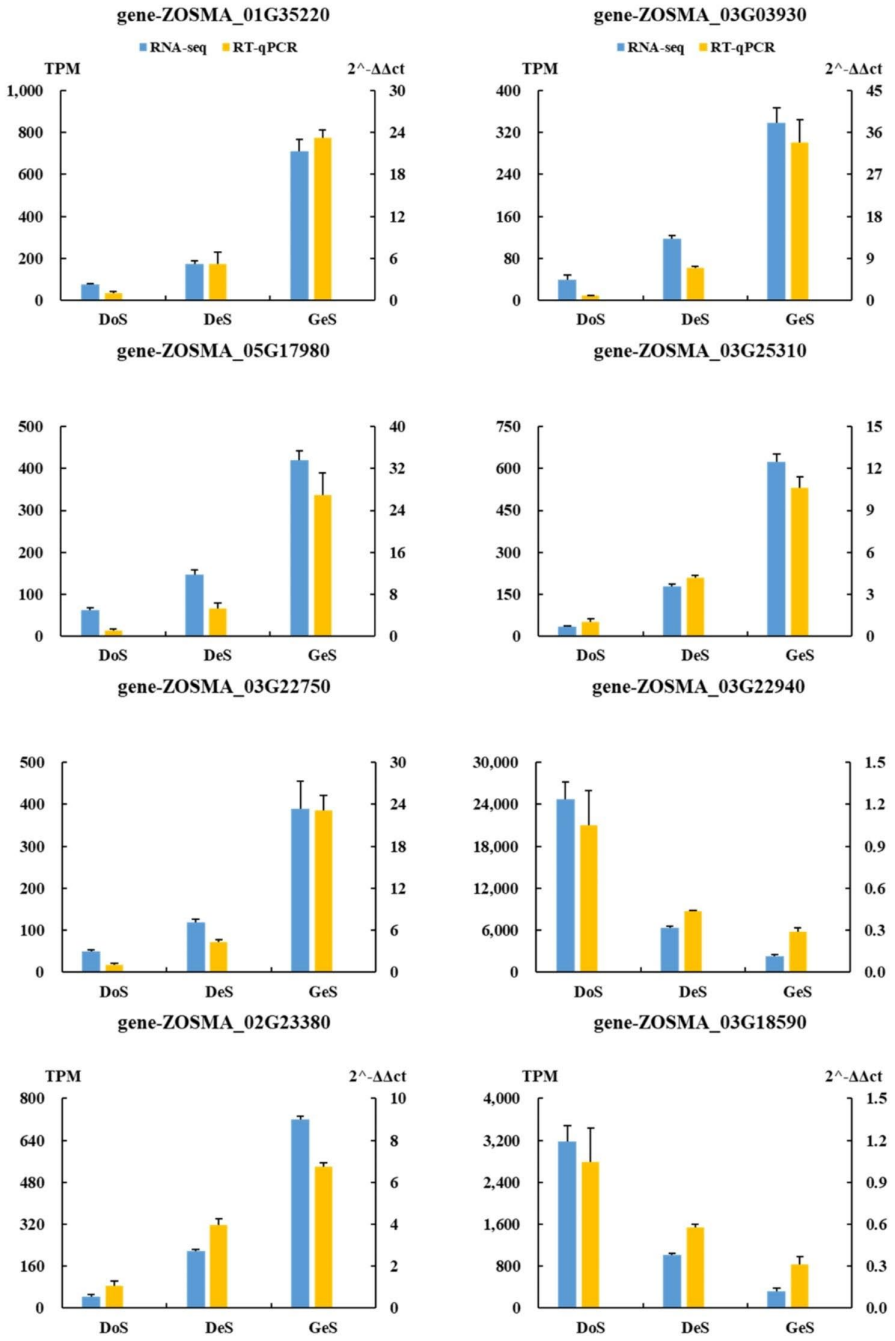
Validation of RNA-seq results by RT-qPCR. Values are the mean ± standard error (SE). N = 3

Transcriptome sequencing of 12 samples (seeds from 3 different states, with 4 replicates in per state, Fig. 1) was completed, with a total of 76.77 Gb clean data obtained. The percentage of Q30 bases was > 90.94%. Clean reads were compared with the designated reference genome for sequence alignment, with the comparison ratio ranging from 83.6 to 95.07%. Principal Component Analysis cluster analysis showed that samples from the same treatment were clustered together but clearly separated from other samples of different treatments, indicating large differences among the three treatments (Fig. 2A). A total of 19,526 expressed genes were detected, including 17,332 known genes and 2,194 novel genes. There were 1,787 up-regulated DEGs and 1,401 down-regulated DEGs for DeS when compared with DoS, and 1,732 up-regulated DEGs and 436 down-regulated DEGs for GeS when compared with DeS (Fig. 2C). RT-qPCR results showed that the gene expression trends were consistent with RNA sequencing data, validating the overall transcriptome profile (Fig. 3). Quantitative 4D-label-free quantitative proteomic analysis was performed with the same samples used for transcriptional profiling, and 165,060 spectrum and 2,220 proteins were identified. PCA analysis showed that differences within groups were minor but significant among the three treatments (Fig. 2B). There were 129 up-regulated DEPs and 49 down-regulated DEGs for DeS when compared with DoS, and 130 up-regulated DEGs and 108 down-regulated DEPs for GeS when compared with DeS (Fig. 2D). DEGs and DEPs were analyzed in combination, and 45 DEGs/DEPs were common between DoS and DeS (Fig. 2E), including 30 co-upregulated DEGs/DEPs and six co-downregulated DEGs/DEPs (Fig. 2G); 33 DEGs/DEPs were common between DeS and GeS (Fig. 2F), including 19 co-upregulated DEGs/DEPs and two co-downregulated DEGs/DEPs (Fig. 2H).

### Comparative transcriptome and proteome analyses

Comparative transcriptome and proteome analyses were performed for DoS vs. DeS and DeS vs. GeS, with a view to understand changes that occurred in eelgrass seeds during the pre-germination (DoS vs. DeS) and germination stages (DeS vs. GeS). Considering that seed germination is the reactivation of multiple physiological metabolic activities, we focused on the metabolic pathways involved in the upregulated genes.

#### Activation of plant signal transduction pathways stimulated by low salinity

Low salinity stimulates eelgrass seed germination, which involved in plant signal transduction systems. Therefore, the present study focused on pathways of Phosphatidylinositol signaling system (map04070) and MAPK signaling pathway-plant (map04016). In the pre-germination stage, KEGG pathway enrichment analysis of upregulated DEGs revealed that both pathways contained some genes that were upregulated significantly, such as phospholipase C, calmodulin (CaM), and MAPKK (Table 1), although the two pathways were not significantly enriched (p > 0.05). In the germination stage, the two pathways were enriched significantly (p < 0.05), with more upregulated DEGs, such as phospholipase C, CaM, MAPK, MAPKK, and MAPKKK (Table 2). We further analyzed the proteomic analysis results corresponding to the transcriptome analysis results. In the pre-germination stage, MAPKK (Zosma03g19400) was upregulated DEP. There was significant upregulation (p < 0.05) of two CaMs, with FC values of 1.25 and 1.40, respectively. Phospholipase C was upregulated but not significantly. In the germination stage, the proteins corresponding to phospholipase C, CaM, MAPK, MAPKK, and MAPKKK exhibited up-regulation trends, although they were not significant.

**Table 1:** Upregulated DEGs (DoS vs. DeS) in pathway phosphatidylinositol signaling system (map04070) and MAPK signaling pathway-plant (map04016)

Gene name	Gene description	Log2FC (DeS/DoS)	Pvalue	Significant	Regulate
**Phosphatidylinositol signaling system (map04070)**					
Zosma03g02710	Inositol-tetrakisphosphate 1-kinase (ITPK1)	1.72	8.75E-58	yes	up
Zosma02g18430	Calmodulin (CALM)	1.40	3.18E-27	yes	up
Zosma04g21070	Inositol-phosphate phosphatase	1.27	1.19E-18	yes	up
Zosma01g32830	Calmodulin (CALM)	1.26	8.52E-17	yes	up
Zosma06g09120	Calmodulin (CALM)	1.20	1.47E-14	yes	up
Zosma03g33450	Chaperonin	1.87	1.79E-14	yes	up
Zosma04g01770	Diacylglycerol kinase 2	1.37	1.11E-09	yes	up
Zosma01g17400	Phospholipase C	1.46	2.18E-04	yes	up
Zosma02g24220	DNAse I-Like superfamily protein	1.07	7.94E-03	yes	up
Zosma04g21910	Diacylglycerol kinase 5-related	1.01	1.79E-02	yes	up
**MAPK signaling pathway-plant (map04016)**					
Zosma02g18430	Calmodulin (CALM)	1.40	3.18E-27	yes	up
Zosma01g32830	Calmodulin (CALM)	1.26	8.52E-17	yes	up
Zosma05g11640	Serine/threonine-protein kinase SRK2 (SNRK2)	1.33	2.08E-15	yes	up
Zosma06g09120	Calmodulin (CALM)	1.20	1.47E-14	yes	up
Zosma03g25670	LRR receptor serine/threonine-protein kinase erecta	3.75	7.18E-08	yes	up
Zosma03g19400	Mitogen-activated protein kinase kinase 4/5, plant (MKK4_5P)	1.06	7.51E-06	yes	up
Zosma01g07670	EIN3-binding F-box protein (EBF1_2)	1.66	1.26E-03	yes	up

**Table 2:** Upregulated DEGs (DeS vs. GeS) in pathway phosphatidylinositol signaling system (map04070) and MAPK signaling pathway-plant (map04016)

Gene name	Gene description	Log2FC (GeS/DeS)	Pvalue	Significant	Regulate
**Phosphatidylinositol signaling system (map04070)**					
Zosma06g17200	Calcium-binding protein CML30-related	5.95	7.71E-96	yes	up
Zosma01g17380	Phosphoinositide phospholipase C	2.11	3.86E-74	yes	up
Zosma06g21480	Phosphatidylinositol phospholipase C, delta (PLCD)	1.60	8.04E-68	yes	up
Zosma06g22670	Inositol-phosphate phosphatase	2.39	9.23E-43	yes	up
Zosma04g23450	1-Phosphatidylinositol-3-phosphate 5-kinase	1.86	2.37E-24	yes	up
Zosma06g25910	Calcium-binding protein CML (CML)	1.85	6.92E-21	yes	up
Zosma02g16920	Phosphatidylinositol 4-phosphate 5-kinase 1-related	1.25	2.85E-17	yes	up
Zosma02g04740	1-Phosphatidylinositol-3-phosphate 5-kinase (PIKFYVE, FAB1)	1.05	5.80E-16	yes	up
Zosma01g17310	Phosphoinositide phospholipase C	1.14	2.88E-12	yes	up
Zosma01g17240	Phosphoinositide phospholipase C	4.00	6.01E-12	yes	up
Zosma01g17400	Phospholipase C	1.43	5.68E-08	yes	up
Zosma04g21910	Diacylglycerol kinase 5-related	1.20	6.60E-06	yes	up
Zosma01g12110	EF-Hand calcium-binding domain containing protein	2.15	6.72E-06	yes	up
**MAPK signaling pathway-plant (map04016)**					
Zosma05g03810	WRKY transcription factor 22	4.75	4.85E-166	yes	up
Zosma06g17200	Calcium-binding protein CML30-related	5.95	7.71E-96	yes	up
Zosma06g09020	Protein phosphatase 2 C	2.70	2.19E-74	yes	up
Zosma04g23910	Transcription factor VIP1	1.95	2.04E-64	yes	up
Zosma03g01510	Mitogen-activated protein kinase 1/3 (MAPK1_3)	1.73	3.67E-54	yes	up
Zosma06g26970	WRKY transcription factor 25-related	4.25	1.15E-52	yes	up
Zosma01g16060	Protein phosphatase 2 C (PP2C)	2.97	1.84E-26	yes	up
Zosma02g01750	VQ motif	2.96	3.48E-26	yes	up
Zosma02g07340	WRKY transcription factor 1-related	4.48	9.39E-25	yes	up
Zosma06g25910	Calcium-binding protein CML (CML)	1.85	6.92E-21	yes	up
Zosma02g23050	Respiratory burst oxidase (RBOH)	5.09	1.31E-14	yes	up
Zosma03g19400	Mitogen-activated protein kinase kinase 4/5, plant (MKK4_5P)	1.04	8.35E-13	yes	up
Zosma03g14010	Mitogen-activated protein kinase	5.96	6.31E-09	yes	up
Zosma03g00810	Mitogen-activated protein kinase kinase	7.57	3.71E-08	yes	up
Zosma01g03460	Mitogen-activated protein kinase kinase	3.27	1.57E-07	yes	up
Zosma06g01320	Abscisic acid receptor PYL1-related	1.37	1.98E-06	yes	up
Zosma03g35940	Nucleoside-diphosphate kinase (ndk, NME)	2.15	6.45E-06	yes	up
Zosma01g12110	EF-Hand calcium-binding domain containing protein	2.15	6.72E-06	yes	up
Zosma05g07070	AGC (CAMP-dependent, CGMP-dependent and protein kinase C) kinase family protein-related	1.74	1.10E-05	yes	up
Zosma04g05340	Mitogen-activated protein kinase	1.09	1.27E-04	yes	up
Zosma05g10360	Transcription factor MYC3-related	5.38	5.98E-04	yes	up

#### Mobilization of main reserves in eelgrass seed

In the pre-germination stage, KEGG pathway enrichment analysis was performed on the upregulated DEGs (Table 3). Among the secondary pathways, tricarboxylic acid (TCA) cycle, amino sugar and nucleotide sugar metabolism, and glycolysis/gluconeogenesis were significantly enriched in carbohydrate metabolism; in addition, starch and sucrose metabolism, fructose and mannose metabolism, also had several upregulated genes, such as sucrose synthase, β-glucosidase, and phosphofructokinase. The results indicated that the breakdown and utilization of starch and sugars provided energy and carbon sources in the early stages of germination. Proteasome was the significantly upregulated enrichment pathway, indicating that the ubiquitin/proteasome pathway was involved in protein mobilization and degradation during early seed germination, where the genes enriched either belong to the 20 S proteasome system or the 26 S proteasome system. In addition, the fatty acid biosynthesis pathway was enriched significantly, suggesting that the breakdown of storage lipids was also a critical step in the early stages of germination. Ribosome and protein processing in endoplasmic reticulum were the two most significantly enriched pathways for in genetic information processing, with the highest number of DEGs, indicating that the translation and processing of the protein was significantly activated. Correspondingly, proteomic analysis identified 16 upregulated DEPs involved in carbohydrate metabolism (Table S1). Furthermore, Acetyl-CoA carboxylase and Long-Chain Acyl-CoA Synthetase in fatty acid biosynthesis were upregulated, although the differences were not significant (1 < FC < 1.5 and p > 0.05). None of the up-regulated DEPs were found in the proteasome pathway; however, a total of 20 proteins with upregulation trends were identified, with the vast majority of proteins belonging to the 26 S proteasome system, suggesting that the 26 S proteasome system played a major role in the degradation of stored proteins.

**Table 3:** KEGG pathway enrichment analysis of upregulated differentially expressed genes (DEGs) set (DoS vs. DeS)

Gene Num	Pathway id	Description	Pvalue	First category	Second category
18	map00630	Glyoxylate and dicarboxylate metabolism	1.49E-04	Metabolism	Carbohydrate metabolism
13	map00020	Citrate cycle (TCA cycle)	2.92E-04	Metabolism	Carbohydrate metabolism
23	map00520	Amino sugar and nucleotide sugar metabolism	6.30E-04	Metabolism	Carbohydrate metabolism
20	map00010	Glycolysis / Gluconeogenesis	7.44E-03	Metabolism	Carbohydrate metabolism
9	map00640	Propanoate metabolism	4.30E-02	Metabolism	Carbohydrate metabolism
3	map00072	Synthesis and degradation of ketone bodies	3.35E-03	Metabolism	Lipid metabolism
10	map00061	Fatty acid biosynthesis	9.46E-03	Metabolism	Lipid metabolism
9	map00260	Glycine, serine and threonine metabolism	4.81E-02	Metabolism	Amino acid metabolism
6	map00460	Cyanoamino acid metabolism	1.23E-02	Metabolism	Metabolism of other amino acids
15	map00900	Terpenoid backbone biosynthesis	4.35E-06	Metabolism	Metabolism of terpenoids and polyketides
2	map00909	Sesquiterpenoid and triterpenoid biosynthesis	2.62E-02	Metabolism	Metabolism of terpenoids and polyketides
57	map03010	Ribosome	2.40E-07	Genetic Information Processing	Translation
33	map04141	Protein processing in endoplasmic reticulum	4.35E-05	Genetic Information Processing	Folding, sorting and degradation
12	map03050	Proteasome	1.83E-02	Genetic Information Processing	Folding, sorting and degradation
7	map04130	SNARE interactions in vesicular transport	2.17E-02	Genetic Information Processing	Folding, sorting and degradation
26	map04145	Phagosome	1.22E-07	Cellular Processes	Transport and catabolism
25	map04144	Endocytosis	2.34E-02	Cellular Processes	Transport and catabolism

In the germination stage, KEGG pathway enrichment analysis was performed on the upregulated DEG set. In total, 24 KEGG pathways were enriched significantly for the upregulated genes, with 19 KEGG pathways falling under metabolism (Table 4). Among the secondary pathways, starch and sucrose metabolism, galactose metabolism, amino sugar and nucleotide sugar metabolism, fructose and mannose metabolism, pentose phosphate pathway, and glycolysis/gluconeogenesis were significantly enriched pathways in carbohydrate metabolism. Fatty acid elongation, linoleic acid metabolism, and α-linolenic acid metabolism were significantly enriched pathways in lipid metabolism. The results indicated that carbohydrate metabolism and lipid metabolism processes were continuously activated during seed germination to maintain the mobilization of reserves during germination. Correspondingly, in the case of the proteomics results (Table S2), six upregulated DEPs were found in the significantly enriched carbohydrate metabolism pathways, and one DEP (Allene oxide synthase, Zosma01g01290) was upregulated in the significantly enriched lipid metabolism pathways.

**Table 4:** KEGG pathway enrichment analysis of upregulated differentially expressed genes (DEGs) (DeS vs. GeS)

Gene Num	Pathway id	Description	Pvalue	First category	Second category
20	map00500	Starch and sucrose metabolism	3.53E-04	Metabolism	Carbohydrate metabolism
11	map00052	Galactose metabolism	3.92E-04	Metabolism	Carbohydrate metabolism
19	map00520	Amino sugar and nucleotide sugar metabolism	7.60E-04	Metabolism	Carbohydrate metabolism
12	map00051	Fructose and mannose metabolism	1.41E-03	Metabolism	Carbohydrate metabolism
10	map00030	Pentose phosphate pathway	5.44E-03	Metabolism	Carbohydrate metabolism
16	map00010	Glycolysis / Gluconeogenesis	1.07E-02	Metabolism	Carbohydrate metabolism
11	map00562	Inositol phosphate metabolism	1.41E-02	Metabolism	Carbohydrate metabolism
9	map00073	Cutin, suberine and wax biosynthesis	1.93E-05	Metabolism	Lipid metabolism
8	map00062	Fatty acid elongation	1.43E-03	Metabolism	Lipid metabolism
6	map00591	Linoleic acid metabolism	1.84E-03	Metabolism	Lipid metabolism
5	map00565	Ether lipid metabolism	2.81E-02	Metabolism	Lipid metabolism
8	map00592	alpha-Linolenic acid metabolism	6.62E-03	Metabolism	Lipid metabolism
8	map00350	Tyrosine metabolism	1.20E-02	Metabolism	Amino acid metabolism
7	map00360	Phenylalanine metabolism	2.21E-02	Metabolism	Amino acid metabolism
21	map00940	Phenylpropanoid biosynthesis	8.49E-05	Metabolism	Biosynthesis of other secondary metabolites
10	map00941	Flavonoid biosynthesis	3.04E-03	Metabolism	Biosynthesis of other secondary metabolites
5	map00905	Brassinosteroid biosynthesis	4.71E-03	Metabolism	Metabolism of terpenoids and polyketides
6	map00920	Sulfur metabolism	2.35E-02	Metabolism	Energy metabolism
10	map00480	Glutathione metabolism	4.39E-02	Metabolism	Metabolism of other amino acids
21	map04016	MAPK signaling pathway - plant	4.96E-05	Environmental Information Processing	Signal transduction
13	map04070	Phosphatidylinositol signaling system	2.65E-03	Environmental Information Processing	Signal transduction
37	map04626	Plant-pathogen interaction	1.52E-07	Organismal Systems	Environmental adaptation
11	map04712	Circadian rhythm - plant	2.98E-03	Organismal Systems	Environmental adaptation
5	map04933	AGE-RAGE signaling pathway in diabetic complications	8.16E-03	Human Diseases	Endocrine and metabolic disease

**Fig. 4:**
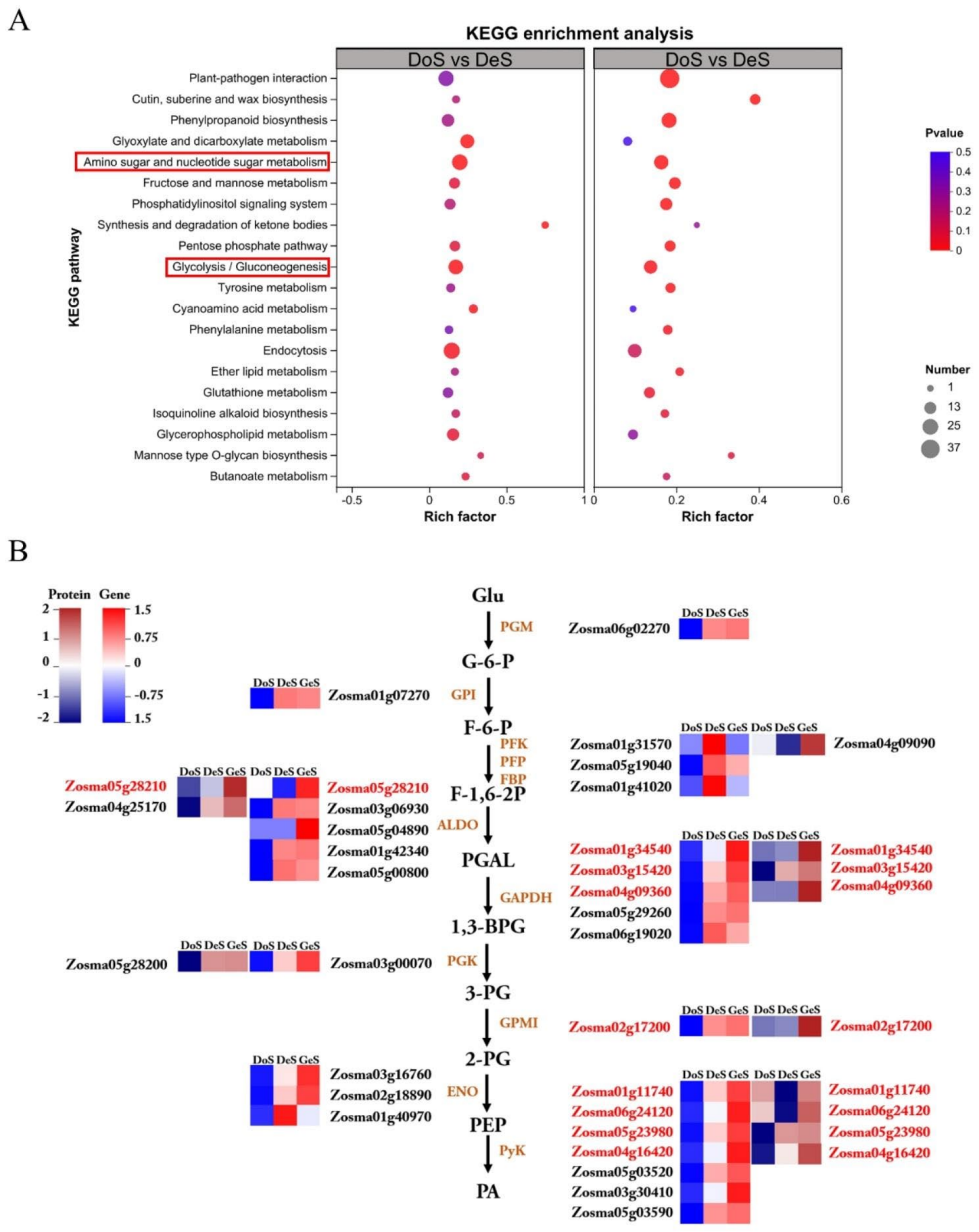
Conjoint analysis of pre-germination and germination stages. (**a**) Kyoto Encyclopedia of Genes and Genomes (KEGG) results for two stages of upregulated DEGs sets. The ordinate represents the pathway name, and the abscissa is the enrichment factor, the larger the enrichment factor, the greater the degree of enrichment, the size of the circle indicates the number of genes enriched in the pathway, the circle color represents the p value. (**b**) Upregulated genes and proteins involved in glycolysis pathway, the red font indicates the same genes identified by both transcriptome and proteome

Conjoint analysis of KEGG results for both stages of upregulated gene sets revealed that amino sugar and nucleotide sugar metabolism and glycolysis/ gluconeogenesis were KEGG pathways enriched significantly in both stages (Fig. 4A, p < 0.05), indicating that the two processes played a substantial role in the eelgrass seed germination process. The upregulated genes and proteins involved in glycolysis pathway were further visualized (Fig. 4B).

#### Plant hormone signal transduction pathway dynamics

The genes and proteins identified in the plant hormone signal transduction pathway (map04075) were analyzed, in addition to the key genes and proteins involved in synthesis, catabolism, and signal transduction of abscisic acid (ABA) and gibberellin (GA).

In the pre-germination stage, 21 DEGs and seven proteins were identified in map04075 (Table S3), with the expression of mitogen-activated protein kinase kinase 4/5, plant (MKK4_5P) at the transcriptome level being upregulated significantly and being consistent with that proteomic level. In the germination stage, 28 DEGs and seven proteins were identified in map04075 (Table S4).

The ABA-related genes and proteins were analyzed. In the pre-germination stage, two DEGs related to ABA synthesis and three DEGs positively regulating ABA signaling were significantly down-regulated, and four DEGs negatively regulating ABA signaling were significantly up-regulated (Table 5). None of the DEPs related to ABA were identified. In addition, two PP2C proteins were upregulated although not significantly. In the germination stage, 19 DEGs negatively regulating ABA signaling were significantly up-regulated, and two DEGs positively regulating ABA signaling were significantly down-regulated (Table 5). In total, three DEPs were identified in the proteome, but including two down-regulated PP2C proteins. It is worth that one PP2C protein was upregulated significantly (p < 0.05), although the FC was less than the defined threshold of 1.35 < 1.5. In addition, allene oxide synthase (Zosma01g01290, AOS), an important enzyme in jasmonic acid synthesis, was found to be a significantly upregulated protein in 2.2.2 lipid mobilization analysis.

**Table 5:** Differentially expressed genes (DEGs) identified involved in abscisic acid (ABA) regulation in the pre-germination stage (DoS vs. DeS) and the germination stage (DeS vs. GeS)

Gene name	Gene description	Log2FC (DeS/DoS or GeS/DeS)	Pvalue	Significant	Regulate	Function
**DoS vs. DeS**						
Zosma03g34840	Zeaxanthin epoxidase (ZEP, ABA1)	-2.39	2.12E-02	yes	down	ABA synthesis
Zosma01g01740	Carotenoid cleavage dioxygenase (CCD)	-1.27	7.12E-17	yes	down	
Zosma02g18130	Abscisic acid insensitive 5-like protein 4	-1.08	2.61E-20	yes	down	Positively regulating ABA signaling
Zosma04g24260	ABA responsive element binding factor (ABF)	-1.09	6.18E-04	yes	down	
Zosma05g03420	Abscisic acid insensitive 5	-1.44	1.66E-29	yes	down	
Zosma05g04480	Protein phosphatase 2 C 61-correlated	1.36	9.21E-36	yes	up	Negatively regulating ABA signaling
Zosma05g15080	Protein phosphatase 2 C 33-correlated	1.16	1.05E-11	yes	up	
Zosma06g16930	Protein phosphatase 2 C 10-correlated	1.52	5.50E-11	yes	up	
Zosma01g03800	Protein phosphatase 2 C 33-correlated	1.73	2.78E-07	yes	up	
**DeS vs. GeS**						
Zosma04g24260	ABA responsive element binding factor (ABF)	-1.29	3.95E-04	yes	down	Positively regulating ABA signaling
Zosma04g25480	Abscisic acid receptor PYR/PYL family (PYL)	-1.79	2.42E-23	yes	down	
Zosma02g21670	Protein phosphatase 2 C-like protein-related	6.75	8.67E-135	yes	up	Negatively regulating ABA signaling
Zosma06g22190	Protein phosphatase 2 C 63-related	5.84	1.47E-07	yes	up	
Zosma01g02300	Protein phosphatase 2 C 27-related	4.86	5.56E-03	yes	up	
Zosma02g21130	Protein phosphatase 2 C 36-related	3.50	5.40E-53	yes	up	
Zosma01g36660	Protein phosphatase 2 C-like protein-related	3.07	7.01E-48	yes	up	
Zosma01g16060	Protein phosphatase 2 C	2.97	1.84E-26	yes	up	
Zosma01g25210	Protein phosphatase 2 C 36-related	2.97	4.21E-15	yes	up	
Zosma06g09020	Protein phosphatase 2 C 16-related	2.70	2.19E-74	yes	up	
Zosma05g28040	Protein phosphatase 2 C	2.06	3.73E-39	yes	up	
Zosma01g03800	Protein phosphatase 2 C 33-related	1.96	3.64E-41	yes	up	
Zosma04g25340	Protein phosphatase 2 C 12-related	1.94	2.84E-35	yes	up	
Zosma05g31280	Protein phosphatase 2 C 46-related	1.46	7.53E-35	yes	up	
Zosma06g16930	Protein phosphatase 2 C 10-related	1.24	1.12E-15	yes	up	
Zosma05g15080	Protein phosphatase 2 C 33-related	1.21	4.81E-23	yes	up	
Zosma04g22810	Protein phosphatase 2 C 68-related	1.21	1.42E-11	yes	up	
Zosma06g28610	Protein phosphatase 2 C homolog 2/3	1.21	3.23E-11	yes	up	
Zosma03g21390	Protein phosphatase 2 C 46-related	1.11	4.23E-18	yes	up	
Zosma01g09260	Protein phosphatase 2 C 10-related	1.05	5.50E-11	yes	up	
Zosma05g21020	Protein phosphatase 2 C 20-related	1.03	1.62E-11	yes	up	

The GA related genes and proteins were analyzed. In the pre-germination stage, two DEGs related to GA synthesis and three DEGs related to GA regulation were up-regulated significantly; two DEGs related to GA inactivation were down-regulated (Table 6). In the germination stage, one DEG related to GA synthesis, two DEGs related to GA regulation, and one DEG related to GA signaling were significantly up-regulated (Table 6). Regretfully, none of the DEPs related to GA in both stages were identified.

**Table 6:** Differentially expressed genes (DEGs) identified involved in gibberellins (GA) regulation in the pre-germination stage (DoS vs. DeS) and the germination stage (DeS vs. GeS)

Gene name	Gene description	Log2FC (DeS/DoS or GeS/DeS)	P value	Significant	Regulate	Function
**DoS vs. DeS**						
Zosma06g07970	Ent-kaurene oxidase	3.31	5.94E-04	yes	up	GA synthesis
Zosma05g07360	Gibberellin 3-beta-dioxygenase	2.87	3.23E-12	yes	up	
Zosma04g19670	Gibberellin regulated protein 4	2.72	1.71E-14	yes	up	GA regulation
Zosma02g20910	Gibberellin regulated protein	2.12	1.06E-11	yes	up	
Zosma03g29110	Gibberellin regulated protein 14-related	1.56	2.78E-07	yes	up	
Zosma06g03950	Gibberellin 2-beta-dioxygenase 2-related	-1.70	5.57E-04	yes	down	GA inactivation
Zosma02g18300	Gibberellin 2-beta-dioxygenase 4	-2.29	6.73E-03	yes	down	
**DeS vs. GeS**						
Zosma06g07970	Ent-kaurene oxidase	2.78	3.00E-14	yes	up	GA synthesis
Zosma03g31640	GRAS domain family (GRAS)	3.24	1.48E-36	yes	up	GA signal transduction
Zosma03g29100	Gibberellin regulated protein (GASA)	1.49	4.06E-05	yes	up	GA regulation
Zosma03g29110	Gibberellin regulated protein 14-related	1.23	1.02E-06	yes	up	

#### Analysis of cell wall-related processes

**Fig. 5:**
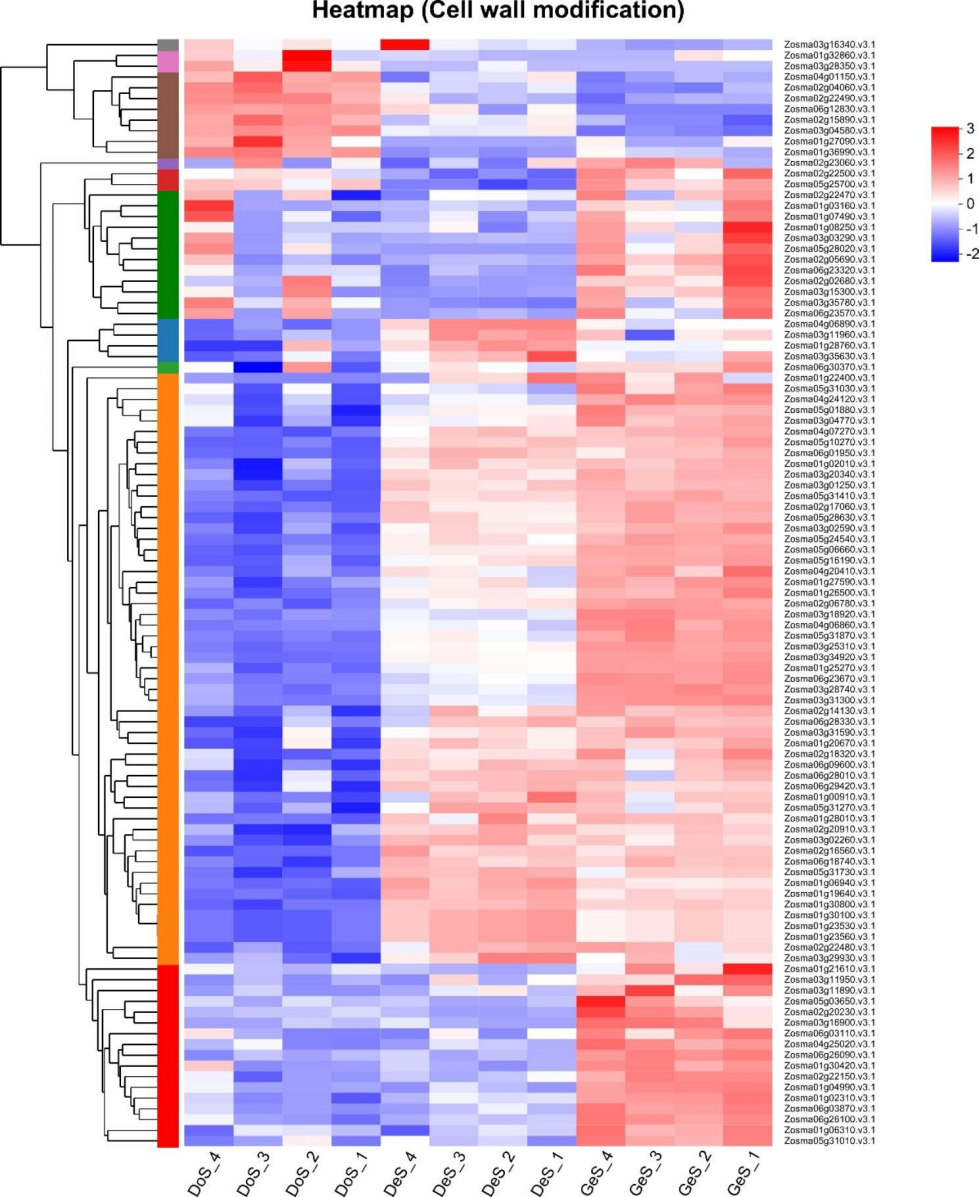
Heat map analysis of genes involved in cell wall modification

Degradation, remodeling, and modification of the cell wall are vital active processes during seed germination. Therefore, we manually collected DEGs associated with the processes in two stages (DoS vs. DeS, DeS vs. GeS); in total, 71 DEGs and 56 DEGs, respectively, were observed in the two stages. The two gene sets were combined for a total of 103 genes, and the heat map analysis is presented in Fig. 5. As the seeds germinated, the cell wall degradation, remodeling, and modification processes were activated gradually and the expression of related genes was upregulated. Similarly, we manually collected DEPs associated with the processes in two stages. Two DEPs were found in DoS vs. DeS, both of which were pectin methylesterase; 10 DEPs were found in DeS vs. GeS, including expansin, alpha-mannosidase, pectin methylesterase, endo-beta-1,4-glucanase, and beta-1,3-endoglucanase. The proteome analysis results were consistent with the transcriptome analysis results.

#### Antioxidant system activation

**Fig. 6:**
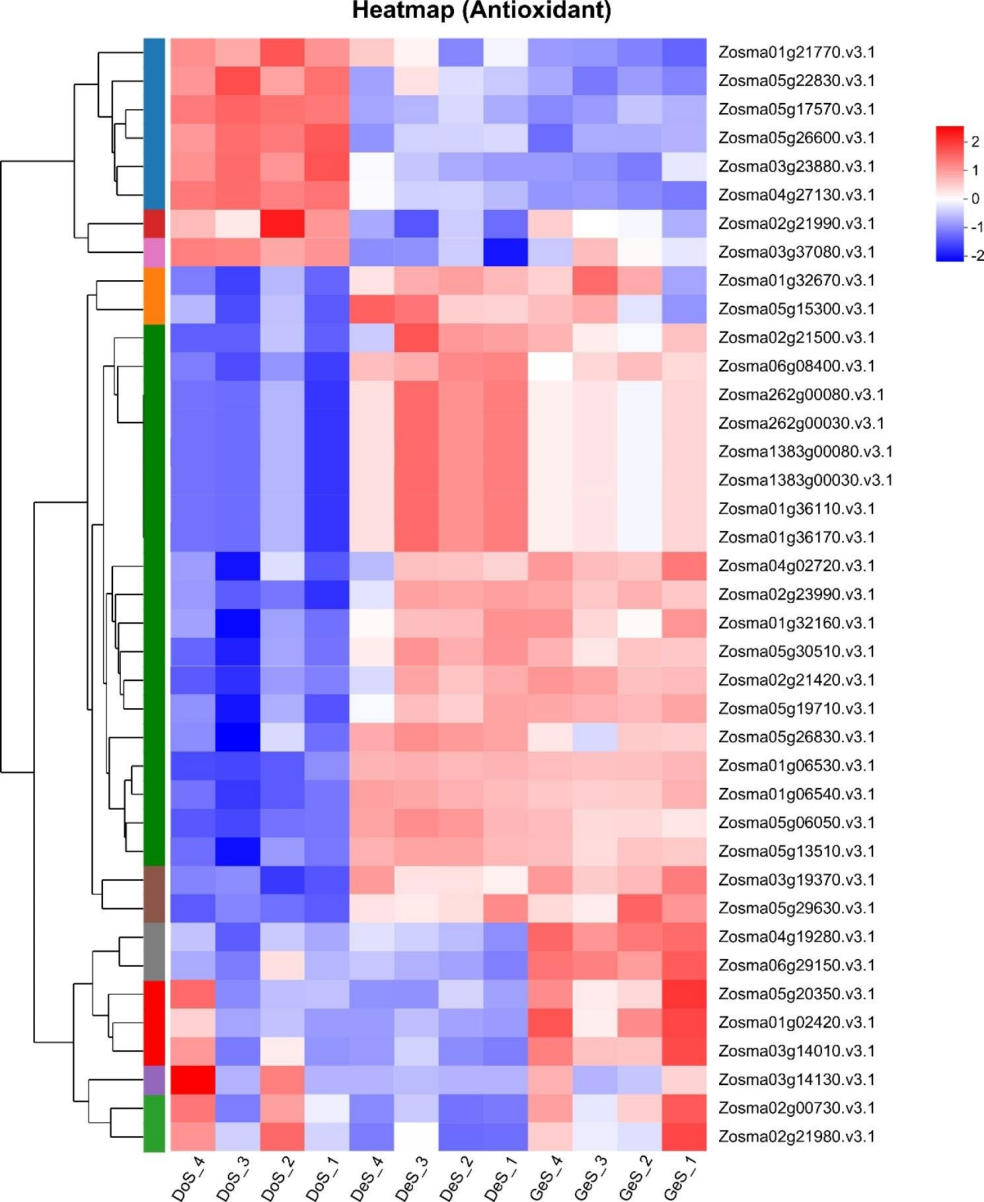
Heat map analysis of genes involved in the antioxidant system

During the activation of metabolic processes following imbibition, reactive oxygen species (ROS) production increases with an increase in germination, which stimulates the activation of antioxidant systems to scavenge excess ROS and maintain intracellular redox homeostasis. We analyzed three GO terms related to the antioxidant process, namely antioxidant activity (GO:0016209), response to oxidative stress (GO:0006979), and cellular response to oxidative stress (GO:0034599). In total, 35 DEGs were found in DoS vs. DeS, out of which 23 were upregulated and 12 were downregulated. Most of the up-regulated antioxidant enzymes were peroxidase, in addition to glutaredoxin, glutathione peroxidase, peroxiredoxin, and methionine sulfoxide reductase. In total, eight DEGs were found in DeS vs. GeS, all of which were up-regulated. All the genes of the two stages were combined for joint analysis (Fig. 6). A number of antioxidant enzymes were activated to prevent damage from oxidative stress in both the pre-germination and germinated stages, and the number of antioxidant enzymes mobilized was higher in the germinated stage. Accordingly, three GO terms (GO:0016209, GO:0006979, and GO:0034599) related to antioxidant processes in the proteome were analyzed. Two upregulated DEPs were found in DoS vs. DeS, namely PLAT/LH2 family protein (related to lipoxygenase) and glutathione peroxidase. Besides, peroxidase, peroxiredoxin, superoxide dismutase, and ascorbate peroxidase exhibited upregulation. No DEPs were upregulated in DeS vs. GeS; however, PLAT/LH2 family protein, peroxiredoxin, peroxidase superfamily protein, and glutathione peroxidase also exhibited upregulation.

### Temporal expression trend analysis

**Fig. 7:**
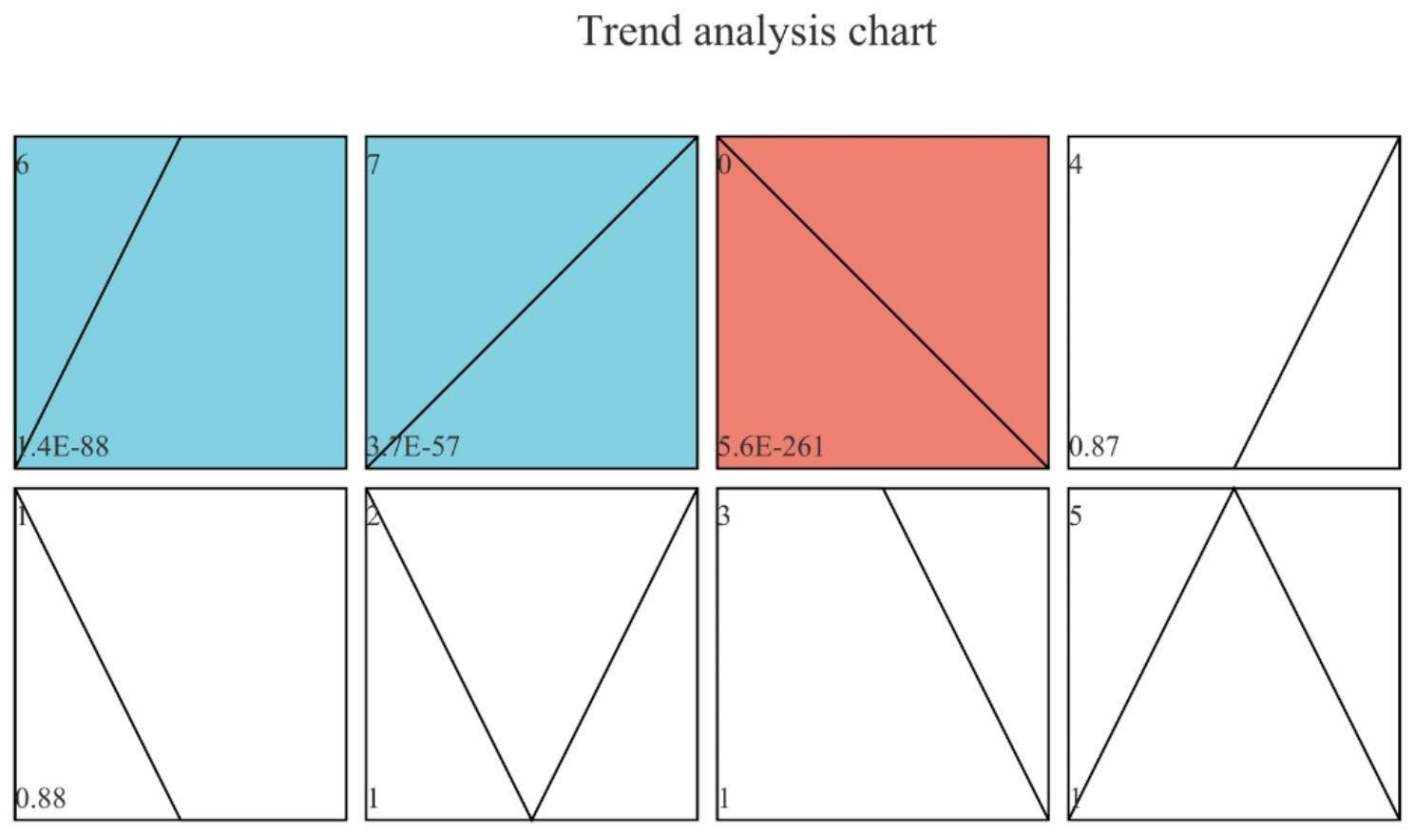
Temporal expression trend analysis chart. Changes in gene expression trends is shown. Each profile corresponds to a rectangle, the number in the upper left corner of the rectangle is the profile number, starting from 0, the broken line is the trend of expression quantity over time, and the value in the lower left corner is its corresponding significance level, p-value. Colored trend charts: Indicate that the temporal pattern of the profile is in line with significant change trend, and the profiles with same color represent belonging to the same cluster (profiles with similar trend are grouped together). Uncolored trend charts: the temporal pattern of profile is in line with non-significant change trend

Temporal expression trend analysis of the transcriptome results was performed at three stages, and three clusters were significantly enriched, including genes that were always up-regulated, genes that were always down-regulated, and genes that were first up-regulated and then remained unchanged (Fig. 7). Further KEGG pathway enrichment analysis was performed on the first two gene sets.

In total, 13 pathways were significantly enriched in the consistently upregulated gene set, with their primary classification being mainly related to Metabolism (10). Carbohydrate metabolism-related pathways accounted for a half of the pathways in the metabolism pathway, including amino sugar and nucleotide sugar metabolism, galactose metabolism, glycolysis/gluconeogenesis, ascorbate and aldarate metabolism, and the pentose phosphate pathway. In addition, ether lipid metabolism in lipid metabolism and phosphatidylinositol signaling system in signal transduction were significantly enriched. In total, eight pathways were enriched in consistently in the downregulated gene set, with their primary classification being mainly related to Genetic Information Processing (4).

### Protein-protein interaction networks analysis of two stages

**Fig. 8:**
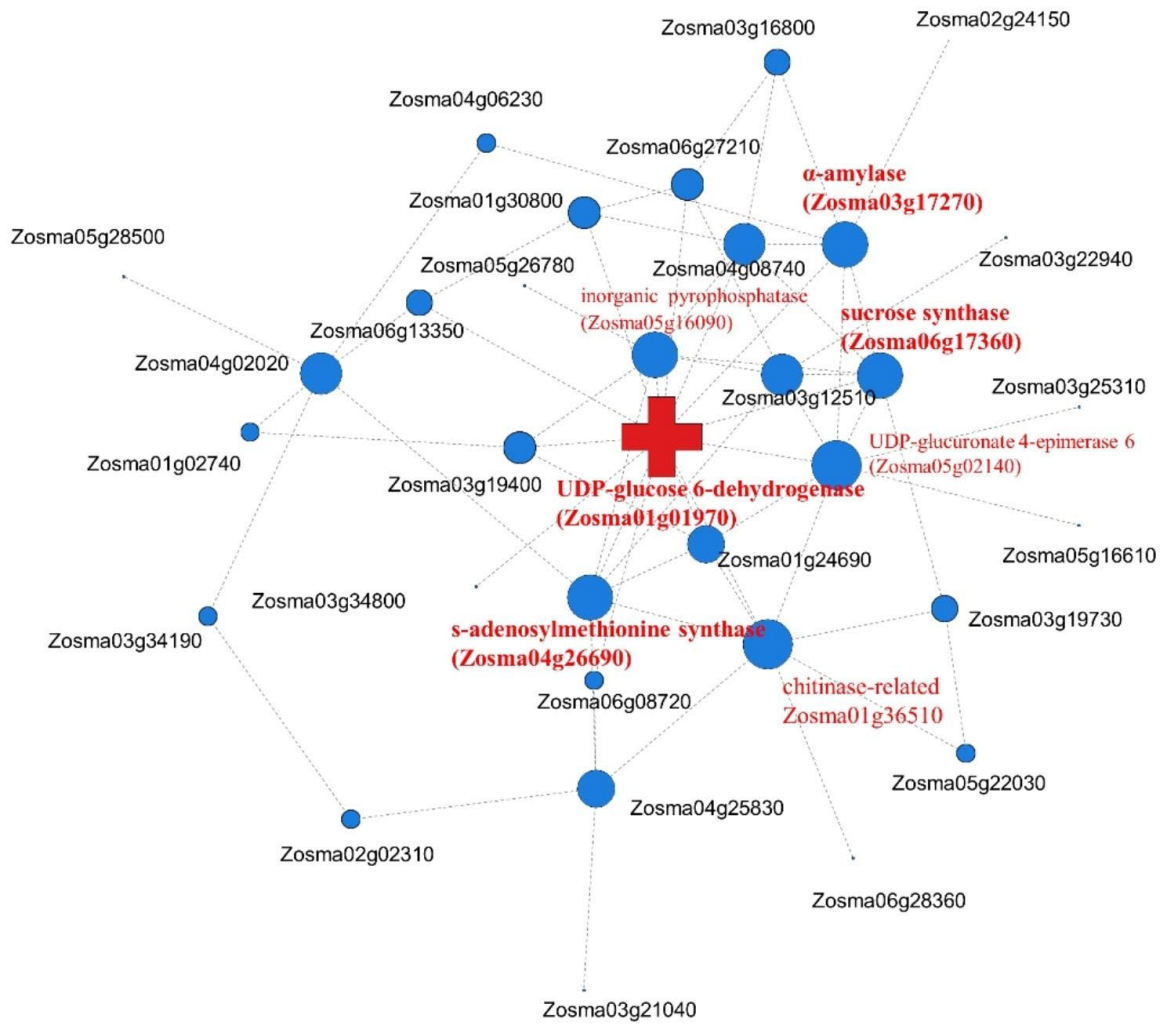
Protein-Protein Interaction network analysis in the pre-germination stage. A node represents a protein, and an edge represents an interaction between two proteins. The size of the node is proportional to the connectivity (degree) of the node, the more edges connected to the node, the greater the connectivity (degree), the larger the node, indicating the greater importance of the node gene in the network

DEGs and DEPs were associated to conduct PPI network analysis. In the pre-germination stage, and UDP-glucose 6-dehydrogenase (UGDH, Zosma01g01970) was at the center of the network, followed by UDP-glucuronate 4-epimerase 6 (Zosma05g02140), chitinase-related (Zosma01g36510), α-amylase (Zosma03g17270), sucrose synthase (Zosma06g17360), s-adenosylmethionine synthase (Zosma04g26690) and inorganic pyrophosphatase (Zosma05g16090) (Fig. 8). PPI analysis did not present a good aggregation result, and the genes were dispersed in the germination stage.

### Transcription factors analysis of two stages

**Fig. 9:**
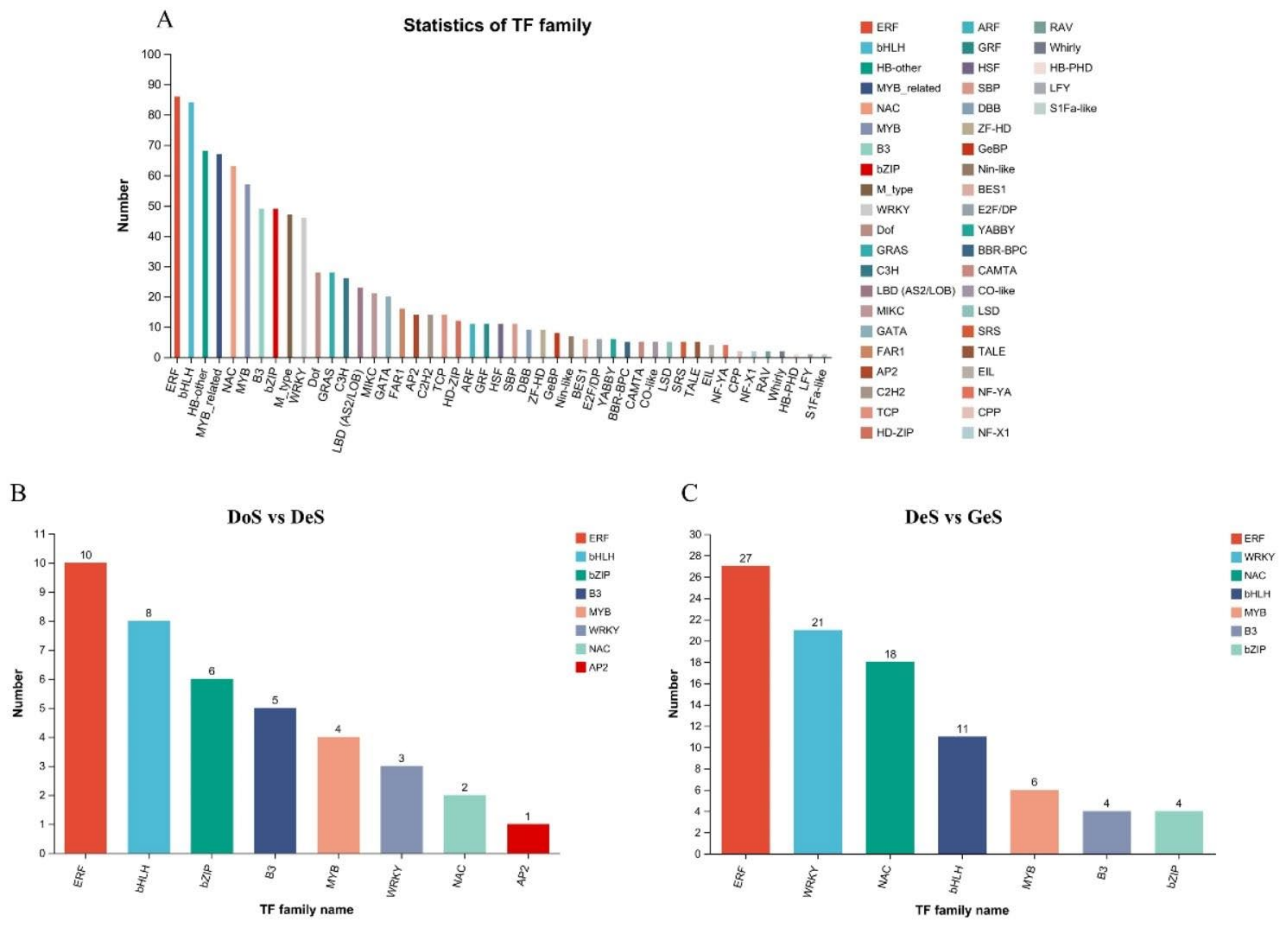
Statistics of transcription factors (TFs). (**a**) Total TFs in all-genes set (**b, c**) TFs in DoS vs. DeS-up differentially expressed gene (DEG) set and DeS vs. GeS-up DEG set

Transcription factor (TF) prediction was performed on the identified genes based on the TF database, PlantTFDB, and a total of 976 transcription factors were predicted, corresponding to 1044 transcripts (Fig. 9A). To identify the key TFs in the pre-germination and germination stages, several major TF families associated with seed dormancy and germination were counted, including MYB, B3, NAC, WRKY, bZIP, and AP2/ERF, in the DoS vs. DeS-up and DeS vs. GeS-up gene sets. Ethylene-responsive TF (ERF) numbers were the highest in the pre-germination period, including eight ERFs, and two dehydration-responsive element binding proteins (DERBs) (Fig. 9B); in the germination stages, ERF, WRKY, and NAC were the top three TFs in terms of number (Fig. 9C).

## Discussion

It has been observed that the increase in freshwater input from precipitation and surface run-off in the spring appears to trigger seed germination in many habitats [29, 34]. Laboratory experiments have also found that low salinity can promote the germination of eelgrass seeds, and this attribute can be applied to eelgrass seedling cultivation by improving the germination rate of eelgrass seeds, which can further be applied to the ecological restoration of eelgrass beds [20, 35, 36]. This study focused on exploring the molecular dynamics behind the germination of eelgrass seeds after low-salinity treatment, and analyzed in detail the key metabolic pathways, in order to increase the understanding of the biology of eelgrass seed germination and providing basic information for future improvement of seagrass breeding.

### Low salinity stimulates the activation of signaling system pathway

According to the results of the present study, during seed germination, genes encoding phospholipase C in the phosphatidylinositol signaling system pathway, genes encoding MAPKKK, MAPKK, and MAPK in the MAPK signaling pathway-plant pathway, and genes encoding CaM that are common to both pathways, were upregulated at both the transcriptional and translational levels. Ca^2+^-dependent signals feature as signal initiators, integrators, or transducers, underpin the regulatory mechanisms that control dormancy alleviation and seed germination [37, 38]. Phosphoinositide/phospholipase C (PI/PLC) signals act upstream of Ca^2+^ signals, and they furnish a cascade of second messenger systems for the induction of Ca^2+^ signals [37], and the PI/PLC pathways were also implicated in the regulation of seed germination via employed by ABA and GA [37, 39, 40]. The MAP kinase cascade reaction consisting of MAPKKK-MAPKK-MAPK, regulated by a second messenger, can amplify and transmit the signal continuously, regulating the expression of the corresponding downstream genes [41, 42].

Based on the above findings, we speculated that when the seeds were dormant, the water did not enter the seeds due to the higher external osmotic pressure under high salinity seawater conditions. In contrast, under low salinity conditions, external water entered the seeds due to osmotic pressure difference, which was similar to the imbibition by dry terrestrial seeds following water absorption, resulting in the activation of intracellular signaling. Thereafter, the signal was cascaded to the nucleus, where the expression of the relevant genes was regulated, in turn initiating subsequent germination-related physiological processes.

### Essential roles of carbohydrates, lipids, and proteins in eelgrass seed germination

The metabolic activity of dormant seeds is attenuated or silent until seed imbibition occurs; afterward, dormancy is broken, germination is activated, and seed stores of starch, protein, and lipids are mobilized actively in the transformation process, allowing subsequent emergence of the radicle and seedling establishment [31, 43]. Studies have shown that eelgrass seed contained on average approximately 50% starch (DW), 10% protein (DW), and 1.3% lipids (DW), with starch being the main form of energy reserve in eelgrass seeds on a weight basis [44, 45]. According to our gene expression data, starch and sucrose metabolism, and fructose and mannose metabolism pathways were enriched with several up-regulated DEGs at both stages, and significant up-regulation of related enzymes occurred at the proteomic level, including amylase, starch synthase, sucrose synthase, and fructokinase. The enzymes hydrolyze starch as well as other sugars into glucose or fructose, which then participate in energy production pathways, such as glycolysis and the TCA cycle, providing sufficient energy for eelgrass seed germination. Comparison of our data with seed germination data for terrestrial plants, such as rice, barley, and quinoa, also revealed similarities, with the upregulation of genes encoding components of sugar, starch, and lipid metabolism [31, 43, 46]. It has been demonstrated that glycolysis was the main source of energy produced by respiration during early germination in some species [47–49]. According to our analysis results, eelgrass seed germination was no exception. Glycolysis was the enriched significantly pathway of persistently upregulated genes in the temporal expression analysis, which indicated that the glycolytic process was activated continuously during eelgrass seed germination and was the main energy source.

Lipids in seeds of higher plants can be used as energy sources during embryonic development [50]. During seed germination, mobilization of stored lipids begins with the breakdown of accumulated triacylglycerols in the oil bodies to free fatty acids and glycerol [51]. Acetyl Co-A produced by fatty acid β oxidation generates succinate in acetaldehyde vesicles via the glyoxylate cycle. Subsequently, succinate is exported to the mitochondria to participate in the TCA cycle, providing the energy and carbon skeleton for subsequent germination and seedling establishment [52]. KEGG pathway enrichment analysis in the present study identified several lipid metabolism-related pathways as significantly upregulated in both stages. In addition, temporal expression analysis identified one lipid metabolism pathway that was consistently upregulated; the results suggested that the breakdown of stored lipids was also a critical step in the germination process of eelgrass seeds. Seed storage proteins are the main source of amino acids in the early stages of seed germination, which can be used for the synthesis of subsequent enzymes and structural proteins, and are also vital for energy production [53–56]. Proteins in soybean seeds are degraded by protease and 26 S proteasome system, whereas rice seeds are degraded by protease during germination [49, 56]. Our combined transcriptomic and proteomic analysis results indicated that the degradation dynamics of storage proteins involved in eelgrass seed germination were similar to those of soybean seeds, implying that the 26 S proteasome system played a major role in their degradation. In addition, seed germination activates various physiological processes, which involve the resynthesis of a large number of enzymes, which could explain the significant activation of protein translation and processing processes.

### GA and ABA-related gene and protein dynamics

Seed dormancy and germination cannot be regulated without phytohormones. ABA and GA have been demonstrated to be the two most critical factors [57]. ABA is associated with the induction and maintenance of dormancy, and GA is associated with seed germination promotion [57]. We analyzed the synthesis, degradation, and signal transduction of ABA and GA. Overall, we observed that some genes involved in ABA synthesis and positive signal transduction were repressed, some genes involved in ABA negative signal transduction were activated; and some genes involved in GA synthesis and positive signal transduction were activated.

Specifically, genes such as ZEP, CCD, ABF, and ABI5 were down-regulated significantly during the pre-germination stage, and PP2C genes were up-regulated significantly in both the pre-germination and germination stages. Based on genetic and functional studies in *Arabidopsis*, key components of the ABA biosynthetic pathway include ZEP/ABA1, which catalyzes the conversion of zeaxanthin to all-trans zeaxanthin, and NCED, belonging to a subfamily of CCD, which cleaves 9-cis xanthophylls to xanthoxin, a precursor of ABA [58, 59]. ABI5 is a bZIP TF and a core TF in ABA signaling that inhibits seed germination and maintains seed dormancy [60, 61]. ABRE binding factor (ABF) TFs regulate the expression of ABRE-dependent genes; therefore, ABF is also a positive regulator of ABA signaling, promoting seed dormancy and inhibiting seed germination [62]. PP2C is one of the core components of ABA signaling; it interacts with SnRK2s, leaving ABA signaling pathway in the “off” state [63, 64]. AOS is an important enzyme in the synthesis of jasmonic acid, which promotes seed germination by inhibiting ABA synthesis and promoting ABA inactivation [65, 66]. In the present study, ZEP and CCD genes were down-regulated significantly and AOS were upregulated significantly, indicating a decrease in ABA synthesis; conversely, the down-regulation of ABF and ABI5 and the up-regulation of PP2C indicated that ABA signaling was inhibited. The two activities resulted in the gradual weakening of dormancy in eelgrass seeds. Studies have shown that GA stimulated germination by inducing hydrolases that weaken barrier tissues, such as the endosperm or seed coat, induce mobilization of seed storage, and embryo expansion [67]. According to our results, the expression levels of KAO and GASA genes were consistently up-regulated during the pre-germination to germination stage; significant up-regulation of GA3-oxidases occurred during the pre-germination stage. KAO is a class of cytochrome P450 monooxygenases in the CYP88A subfamily that catalyzes the conversion of t-kaurenoic acid (KA) to the precursor (GA12) of all GA, thereby determining plant GA concentration [68]. GA3ox catalyzes the final step of the GA biosynthesis pathway to produce active GA molecules [69]. GASA is a class of gene family induced by GA that encodes a small molecular polypeptide [70]. Rubinovich and Weiss [70]. observed that overexpression of the GA-inducible GASA4 gene in *Arabidopsis* promoted the response to GA, thereby facilitating flowering and seed germination.

### Cell wall loosening and remodeling

Endosperm weakening is a crucial stage of the seed germination process. It facilitates radicle breaking through the endosperm and completion of the germination process [71]. Cell Wall Loosening factors (CWLFs) weaken the cell wall by encoding enzymes and non-enzymatic factors, resulting in weakening of the mechanical strength of the cell wall. Enzymatic CWLFs mainly include enzymes encoding the degradation of cellulose, hemicellulose and pectin [72–75]. Nonenzymatic CWLFs mainly include genes encoding expansin and genes related to ROS metabolism [75, 76]. According to our results, a large number of CWLF genes were significantly upregulated in two stages, such as genes encoding cellulose degradation (endoglucanase, beta-glucosidase); genes encoding hemicellulose degradation (beta-mannosidase; beta-galactosidase; beta-xylosidase; XTHs); genes encoding pectin degradation (pectinesterase; polygalacturonase); genes encoding expansin (expansin); and genes encoding ROS (peroxidase). More importantly, several corresponding proteins were significantly upregulated at the proteomic level, and the activation of the genes provided assurance for endosperm to break through the endosperm cell wall, in turn allowing smooth eelgrass seed germination. In addition to CWLFs, some cell wall remodeling enzymes (CWREs), associated with cell wall synthesis, loosening and enhancement, played indispensable roles in seed germination. CWREs generally include the same genes as the enzymatic CWLFs. For example, XTHs, which can also act as cell wall-modifying proteins, are involved in the regulation of embryonic axis or radicle elongation during seed germination [77, 78]. Our findings revealed that the gradual exposure of the cotyledon, as the seed germination process proceeded, implied a continuous process of cell wall degradation, modification, and remodeling, which was reflected in the heat map showing a substantial increase in the number and abundance of the associated genes.

### Activation of antioxidant system

Studies have shown that when resting dry seeds absorb water, their oxygen uptake increased and mitochondrial energy metabolism was reactivated, which provided an important source of ROS [79]. Our analysis revealed that most of the DEGs and proteins identified in both stages were peroxidases, implying that peroxidases may be the main antioxidant enzymes for scavenging ROS in eelgrass during germination. In addition, we observed that lipoxygenase (LOX) as well as peroxiredoxins were important at both the transcriptome and proteome levels. LOX is widely believed to be involved in lipid mobilization during the early stages of seed germination, and facilitates ROS removal during the rapid mobilization of germinating seed reserves to alleviate the oxidative stress [56]. Peroxiredoxin is also a ROS scavenger that protects functional proteins from ROS during seed germination [52, 56]. Based on the heat map analysis, the number of up-regulated genes was greater in germination stage than in pre-germination stage, implying that the mitochondrial metabolic activity was enhanced during the germination process, resulting in greater ROS production.

### HUB genes of the germination process

The results of PPI analysis of DEGs-DEPs at the pre-germination stage showed that the HUB gene at the center of the network was UDP-glucose 6-dehydrogenase (UGDH, Zosma01g01970), α-amylase, sucrose synthase, and s-adenosylmethionine synthase also have higher degree. However, at the germination stage, PPI analysis did not display a good aggregation result and the genes were dispersed. In plants, UGDH is one of the key enzymes involved in amino sugar and nucleotide sugar metabolism and is closely related to polysaccharide biosynthesis [80, 81]. Both amylase and sucrose synthase were significantly up-regulated at the transcriptional and protein levels, suggesting an important role during eelgrass seed germination. Many studies have also shown that starch hydrolases and sugar hydrolases were vital enzymes in the seed germination process [52, 82]. We think that during the germination phase of eelgrass seeds, under the action of amylase and sucrose synthase, the starch stored in the seeds begins to break down and produce ATP and carbohydrate material, which are used not only in seed germination but also in subsequent emergence of the radicle. Methionine metabolism also plays a major role in seed germination [83]. S-adenosylmethionine synthetase (metK) is a key enzyme involved in the synthesis of s-adenosylmethionine (AdoMet), which is a precursor of polyamine, vitamin biotin, and ethylene biosynthesis and provides essential metabolites for DNA synthesis, methylation regulation, and hormone regulation [27]. Our results revealed significant upregulation of metK at both the transcriptome and proteome levels, which is consistent with the characteristic accumulation of metK prior to radicle emergence [83, 84].

### ERF transcription factors in eelgrass seed germination

Our analysis revealed that the ERF family was the most abundant in both stages, and presumably ERF TFs played an important role in the seed germination process. The AP2/ERF (APETALA2/ethylene response factor) structural domain family is a plant-specific [85, 86]. In recent years, several studies have revealed the regulatory mechanism of AP2/ERF-like TFs involved in plant seed germination. Liu and Wu [87] found that an ERF transcription factor isolated from tomato significantly reduced the sensitivity to ABA and auxin during seed germination by inhibiting a key component of the ABA signaling pathway, thereby promoting germination. The following year, Liu et al. [88] also found that ERF transcription factors could promote tomato seed germination through the GA-mediated glucose signaling pathway. Gupta et al. [85] also identified an AP2/ERF TF that negatively regulated ABA responses by altering ABA levels/signaling pathways, thereby promoting seed germination.

## Conclusion

**Fig. 10:**
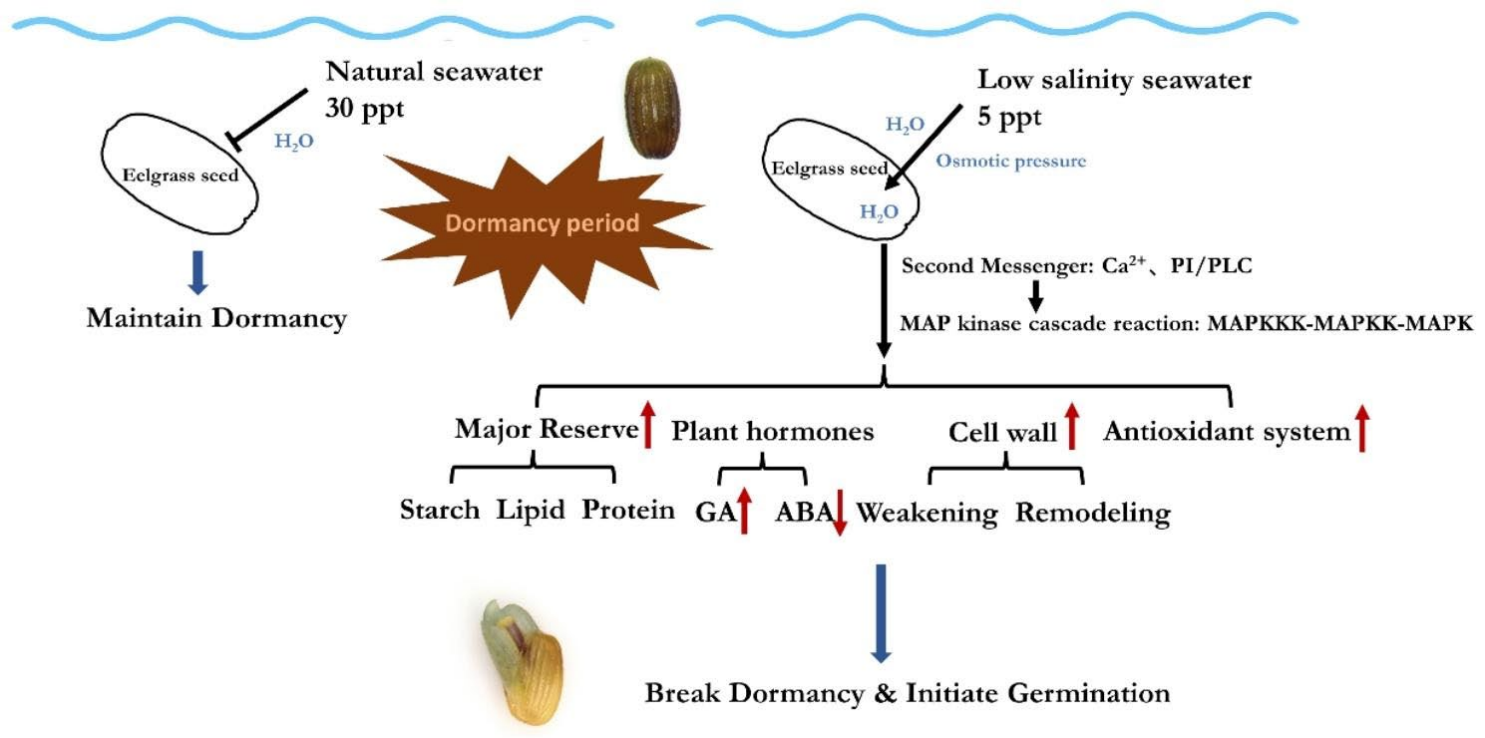
Mechanisms via which low salinity stimulates eelgrass seed germination

In the present study, transcriptomics and proteomics were combined to analyze the regulatory network and the dynamics of key physiological processes in eelgrass seeds at three stages from dormancy to germination under low salinity stimulation (Fig. 10). According to our results, low salinity stimulated the activation Ca^2+^ signaling and phosphatidylinositol signaling, and further initiated various subsequent germination-related physiological processes through the MAPK cascade. Starch, lipids, and storage proteins were actively mobilized to provide energy and substrate for germination; ABA synthesis and signal transduction were inhibited, whereas GA synthesis and signal transduction were activated, weakening seed dormancy and preparing seeds for germination; cell wall weakening and remodeling processes were activated to provide protection for cotyledon emergence. In addition, multiple antioxidant systems were activated to alleviate the oxidative stress generated during the germination process. PPI analysis of DEGs-DEPs of pre-germination revealed that HUB gene at the center of the network was udp-glucose 6-dehydrogenase (UGDH, Zosma01g01970), followed by α-amylase, sucrose synthase, suggesting that the activation of carbohydrate metabolism, such as starch, was essential to support eelgrass seed germination in the early stages. Identification of transcription factors revealed the highest number of ERF TFs in both stages, and it was hypothesized that the ERF family played an active role in the seed germination process. The present study investigated the mechanisms of eelgrass seed germination stimulated by low salinity, and further comprehensively analyzed the dynamics of key physiological processes at the transcriptome and proteome levels, providing experimental data for subsequent in-depth studies of the regulatory mechanisms related to seagrass seed germination.

## Materials and methods

### Experimental design

In this study, eelgrass seeds were collected from their natural habitats, and the collection processes of both conformed to local and national regulations. The voucher specimens of Z. marina were deposited in Marine Biological Museum of the Chinese Academy of Sciences (MBMCAS). The samples were identified by Yi Zhou, a Professor at IOCAS. Specifically, mature eelgrass seeds collected from Swan Lake, Weihai City, Shandong Province, China, in September 2021, and the seed germination season in the area was the following spring [89]. Collected seeds were stored in recirculating seawater tanks (1 m × 1.2 m × 1.5 m) in a laboratory. In December, the seeds are dormant and do not germinate under natural seawater conditions; however, low salinity conditions can promote rapid seed germination. Consequently, in December, 2,000 seeds (four replicates, 500 seeds per replicate) were placed in low salinity seawater with a salinity of 5 ppt; another 1,000 seeds (four replicates, 250 seeds per replicate) were placed in natural seawater with a salinity of 30 ppt. Seeds under both treatments were placed at a temperature of 15 °C and in the dark for germination. After 24 h, dormant seeds (DoS) were collected from the natural seawater group; dehiscent seeds (DeS) and germinated seeds (GeS) were collected from the low salinity seawater group (Fig. 1). The samples were immediately snap-frozen in liquid nitrogen for 15 min after collection, and then stored in a -80 °C refrigerator until the subsequent transcriptomic and proteomic analyses.

### RNA sequencing and RT-qPCR

Seeds from three states (DoS/DeS/GeS, four replicates) were obtained for transcriptome sequencing, and the procedure was as follows: first, using TRIzol® Reagent to extract total RNA from eelgrass seed tissues, then DNase I (TaKara) were used to remove genomic DNA. We select high quality RNA samples to construct subsequent sequencing library. Secondly, we isolated mRNA, and further fragmented it with fragmentation buffer. Then, mRNA was used as templates to synthesize double-stranded cDNA. Subsequently, we performed end-repair, phosphorylation, and “A” base addition to synthesized cDNA, in which the target fragments of 300 bp were selected for libraries, which were then performed for 15 PCR cycles amplification on 2% Low Range Ultra Agarose. Finally, after quantification by TBS380, the Illumina NovaSeq 6000 sequencer (2 × 150-bp read length) were used to sequence the paired-end RNA-seq sequencing library.

To obtain clean reads with high quality, we performed trim and quality control on raw paired-end reads. Afterward, Zostera marina were selected as the reference genome for separately alignment of clean reads to get mapped reads. To identify DEGs (differentially expressed genes) between two different samples, the expression levels of each transcript were calculated according to the transcripts per million reads (TPM) method. RSEM was used to quantify gene abundances. DESeq2 software was used for differential expression analysis. Screening criteria for DEGs were: p-adjust < 0.05 and |log2FC| > 1.

Eight genes were selected for RT-qPCR to validate the transcriptome, with 18 S rRNA as reference gene (Table S5). The above extracted RNA was used as a template for reverse transcription to obtain cDNA. Pre-experimental results showed that the electrophoresis gel of each primer was a single bright band under specific conditions, indicating that there was no specific amplification and the primers were qualified. The RT-qPCR solution included 10 µL of 2× ChamQ SYBR Color qPCR Master Mix, 0.8 µL of both forward and reverse primers (5 μm each), 0.4 µL of 50× ROX Reference Dye, 2 µL of template (cDNA), and 6 µL of ddH2O, made-up to a total volume of 20 µL. Cycling conditions were as follows: the initial step was 95 °C for 5 min, and then 40 cycles (melting at 95 °C for 5 s, annealing at 55 °C for 30 s, and extension at 72 °C for 40 s). Each treatment group had three replicates, and each replicate sample had three multiple pores. The relative expression level was calculated using the 2^−ΔΔCt^ method.

### Proteome analyses

The 4D-label free quantitative proteomic analysis was performed on the same batch of samples (DoS1/2/3/4, DeS1/2/3/4, and GeS1/2/3/4) used for transcription profiling. The detailed operation was as follows: first, the total protein was extracted from the sample; secondly, the concentration of protein supernatant was determined using the Bicinchoninic acid (BCA) method; third, protein samples were subjected to SDS-PAGE (sodium dodecyl sulfate-polyacrylamide gel) electrophoresis analysis to evaluate whether the sample quality met the standards; fourth, the qualified protein samples were treated with reductive alkylation; fifth, an equal amount of protein from each sample was digested with trypsin; sixth, the peptides were desalted and peptide concentrations were determined; seventh, trypsin-digested peptides were analyzed using an EASY nLC-1200 system (Thermo, USA) coupled with a timsTOF Pro2 mass spectrometer (Bruker, Germany) at Majorbio Bio-Pharm Technology Co. Ltd. (Shanghai, China); eighth, MS/MS spectra were searched using MaxQuant v2.0.3.1 software against the protein database. Finally, bioinformatic analysis of proteomic data was performed using the Majorbio Cloud platform (https://cloud.majorbio.com).

P-values and Fold change (FC) for the proteins between the two groups were calculated using R package “t-test”. The thresholds of fold change (> 1.5 or < 0.67) and P-value < 0.05 were used to identify differentially expressed proteins (DEPs). Functional annotation of all identified proteins was performed using GO (http://geneontology.org/) and KEGG pathway (http://www.genome.jp/kegg/). DEPs were further used for Gene Ontology (GO) and Kyoto Encyclopedia of Genes and Genomes (KEGG) enrichment analysis. Protein-protein interaction (PPI) analysis was performed using String v11.5 (https://string-db.org/).

### Electronic supplementary material

Below is the link to the electronic supplementary material.

**Supplementary Material: Table S1.** Upregulated Differentially Expressed Proteins (DEPs) identified in main carbohydrate metabolism pathways in pre-germination stage (DoS vs. DeS). **Table S2.** Upregulated Differentially Expressed Proteins (DEPs) identified in main carbohydrate metabolism and lipid metabolism pathways in the germination stage (DeS vs. GeS). **Table S3.** Differentially Expressed Genes (DEGs) and Differentially Expressed Proteins (DEPs) identified in plant hormone signal transduction pathway (map04075) in the pre-germination stage. **Table S4.** Differentially Expressed Genes (DEGs) and Differentially Expressed Proteins (DEPs) identified in plant hormone signal transduction pathway (map04075) in the germination stage. **Table S5.** Primer sequences of RT-qPCR.

## Data Availability

Transcriptomic sequencing data are available through the NCBI Sequence Read Archive under the accession number PRJNA964694, and the mass spectrometry proteomics data have been deposited to the Proteome Xchange Consortium via the iProX partner repository with the dataset identifier PXD042350.

## References

[1] Cullen-Unsworth L, Unsworth R (2013). Seagrass meadows, ecosystem services, and sustainability. Environment.

[2] Nordlund LM, Unsworth RKF, Gullstrom M, Cullen-Unsworth LC (2018). Global significance of seagrass fishery activity. Fish Fish.

[3] de los Santos CB, Olive I, Moreira M, Silva A, Freitas C, Luna RA, Quental-Ferreira H, Martins M, Costa MM, Silva J, Cunha ME, Soares F, Pousao-Ferreira P, Santos R (2020). Seagrass meadows improve inflowing water quality in aquaculture ponds. Aquaculture.

[4] Neto NC, Pomeroy A, Lowe R, Ghisalberti M (2022). Seagrass meadows reduce wind-wave driven sediment resuspension in a sheltered environment. Front Mar Sci.

[5] Lima MDC, Ward RD, Joyce CB, Kauer K, Sepp K (2022). Carbon stocks in southern England’s intertidal seagrass meadows. Estuar Coast Shelf Sci.

[6] Xu SC, Zhou Y, Qiao YL, Yue SD, Zhang XM, Zhang Y, Liu MJ, Zhang YL, Zhang ZH (2023). Seagrass restoration using seed ball burial in northern China. Restor Ecol.

[7] Waycott M, Duarte CM, Carruthers TJB, Orth RJ, Dennison WC, Olyarnik S, Calladine A, Fourqurean JW, Heck KL, Hughes AR, Kendrick GA, Kenworthy WJ, Short FT, Williams SL (2009). Accelerating loss of seagrasses across the globe threatens coastal ecosystems. Proc Natl Acad Sci U S A.

[8] Zhou Y, Liu P, Liu BJ, Liu XJ, Zhang XM, Wang F, Yang HS (2014). Restoring eelgrass (*Zostera marina* L.) habitats using a simple and effective transplanting technique. PLoS ONE.

[9] Shafer D, Bergstrom P (2010). An introduction to a special issue on large-scale submerged aquatic vegetation restoration research in the Chesapeake Bay: 2003–2008. Restor Ecol.

[10] Paling EI, van Keulen M, Wheeler K, Phillips J, Dyhrberg R (2001). Mechanical seagrass transplantation in Western Australia. Ecol Eng.

[11] van Katwijk MM, Thorhaug A, Marba N, Orth RJ, Duarte CM, Kendrick GA, Althuizen IHJ, Balestri E, Bernard G, Cambridge ML, Cunha A, Durance C, Giesen W, Han QY, Hosokawa S, Kiswara W, Komatsu T, Lardicci C, Lee KS, Meinesz A, Nakaoka M, O’Brien KR, Paling EI, Pickerell C, Ransijn AMA, Verduin JJ (2016). Global analysis of seagrass restoration: the importance of large-scale planting. J Appl Ecol.

[12] Grafnings MLE, Heusinkveld JHT, Hoeijmakers DJJ, Smeele Q, Wiersema H, Zwarts M, van der Heide T, Govers LL (2023). Optimizing seed injection as a seagrass restoration method. Restor Ecol.

[13] Orth RJ, Moore KA, Marion SR, Wilcox DJ, Parrish DB (2012). Seed addition facilitates eelgrass recovery in a coastal bay system. Mar Ecol Prog Ser.

[14] Orth RJ, Lefcheck JS, McGlathery KS, Aoki L, Luckenbach MW, Moore KA, Oreska MPJ, Snyder R, Wilcox DJ, Lusk B (2020). Restoration of seagrass habitat leads to rapid recovery of coastal ecosystem services. Sci Adv.

[15] Olsen JL, Rouze P, Verhelst B, Lin YC, Bayer T, Collen J, Dattolo E, De Paoli E, Dittami S, Maumus F, Michel G, Kersting A, Lauritano C, Lohaus R, Topel M, Tonon T, Vanneste K, Amirebrahimi M, Brakel J, Bostrom C, Chovatia M, Grimwood J, Jenkins JW, Jueterbock A, Mraz A, Stam WT, Tice H, Bornberg-Bauer E, Green PJ, Pearson GA, Procaccini G, Duarte CM, Schmutz J, Reusch TBH, Van de Peer Y (2016). The genome of the seagrass *Zostera marina* reveals angiosperm adaptation to the sea. Nature.

[16] Green EP, Short FT, Frederick T (2003). World Atlas of Seagrasses.

[17] Orth RJ, Harwell MC, Bailey EM, Bartholomew A, Jawad JT, Lombana AV, Moore KA, Rhode JM, Woods HE (2000). A review of issues in seagrass seed dormancy and germination: implications for conservation and restoration. Mar Ecol Prog Ser.

[18] Pan JH, Han HW, Jiang X, Zhang WF, Zhao N, Song SF, Li X, Li XJ (2012). Desiccation, moisture content and germination of *Zostera marina* L. seed. Restor Ecol.

[19] Xu S, Xu SC, Zhou Y, Gu RT, Zhang XM, Yue SD (2020). Long-term seed storage for desiccation sensitive seeds in the marine foundation species *Zostera marina* L. (eelgrass). Glob Ecol Conserv.

[20] Xu SC, Zhou Y, Wang PM, Wang F, Zhang XM, Gu RT (2016). Salinity and temperature significantly influence seed germination, seedling establishment, and seedling growth of eelgrass *Zostera marina* L. PeerJ.

[21] Ridler MS, Dent RC, Arrinton DA (2006). Effects of two Hurricanes on Syringodium filiforme, manatee grass, within the Loxahatchee River Estuary, Southeast Florida. Estuar Coasts.

[22] Steward JS, Virnstein RW, Lasi MA, Morris LJ, Miller JD, Hall LM, Tweedale WA (2006). The impacts of the 2004 Hurricanes on hydrology, water quality, and seagrass in the central Indian river lagoon, Florida. Estuar Coasts.

[23] He DL, Han C, Yao JL, Shen SH, Yang PF (2011). Constructing the metabolic and regulatory pathways in germinating rice seeds through proteomic approach. Proteomics.

[24] Bewley JD (1997). Seed germination and dormancy. Plant Cell.

[25] Xue XF, Jiao FC, Xu HC, Jiao QQ, Zhang X, Zhang Y, Du SY, Xi MH, Wang AG, Chen JT, Wang M (2021). The role of RNA-binding protein, microRNA and alternative splicing in seed germination: a field need to be discovered. BMC Plant Biol.

[26] Nonogaki H, Bassel GW, Bewley JD (2010). Germination-still a mystery. Plant Sci.

[27] Rajjou L, Duval M, Gallardo K, Catusse J, Bally J, Job C, Job D (2012). Seed germination and Vigor. Annu Rev Plant Biol.

[28] Taylor NL. Studies of the development of *Zostera marina* L. Can J Botany. 1957.

[29] Sugiura H, Hiroe Y, Suzuki T, Maegawa M (2009). The carbohydrate catabolism of *Zostera marina* influenced by lower salinity during the pre-germination stage. Fish Sci.

[30] Ma ZH, Bykova NV, Igamberdiev AU (2017). Cell signaling mechanisms and metabolic regulation of germination and dormancy in barley seeds. Crop J.

[31] Hao YQ, Hong YC, Guo HM, Qin PY, Huang AN, Yang XS, Ren GX (2022). Transcriptomic and metabolomic landscape of quinoa during seed germination. BMC Plant Biol.

[32] Huang H, Moller IM, Song SQ (2012). Proteomics of desiccation tolerance during development and germination of maize embryos. J Proteom.

[33] Trindade BMC, Reis RS, Vale EM, Santa-Catarina C, Silveira V (2018). Proteomics analysis of the germinating seeds of *Cariniana Legalis* (Mart.) Kuntze (Meliaceae): an endangered species of the Brazilian Atlantic Rainforest. Braz J Bot.

[34] Phillips RC, Grant WS, McRoy CP (1983). Reproductive strategies of eelgrass (*Zostera-marina* L). Aquat Bot.

[35] Tanner CE, Parham T (2010). Growing *Zostera marina* (eelgrass) from seeds in land-based culture systems for use in restoration projects. Restor Ecol.

[36] Liu YL, Zhang XL, Song W, Wang ZL (2016). Artificial seed germination and seedling production of *Zostera marina* L. by salinity manipulation. Acta Oceanol Sin.

[37] Omoarelojie LO, Kulkarni MG, Finnie JF, van Staden J (2022). Smoke-derived cues in the regulation of seed germination: are Ca^2+^-dependent signals involved?. Plant Growth Regul.

[38] Verma G, Khan S, Agarwal SK, Sharma S (2019). Role of apoplastic calcium during germination and initial stages of seedling establishment in *Vigna radiata* seeds. J Plant Physiol.

[39] Zhang Q, van Wijk R, Shahbaz M, Roels W, Schooten Bv, Vermeer JEM, Zarza X, Guardia A, Scuffi D, Garcia-Mata C, Laha D, Williams P, Willems LAJ, Ligterink W, Hoffmann-Benning S, Gillaspy G, Schaaf G, Haring MA, Laxalt AM, Munnik T (2018). *Arabidopsis* phospholipase C3 is involved in lateral root initiation and ABA responses in seed germination and stomatal closure. Plant Cell Physiol.

[40] Kashem MA, Itoh K, Iwabuchi S, Hori H, Mitsui T (2000). Possible involvement of phosphoinositide-Ca^2+^ signaling in the regulation of alpha-amylase expression and germination of rice seed (*Oryza sativa* L). Plant Cell Physiol.

[41] Mishra NS, Tuteja R, Tuteja N (2006). Signaling through MAP kinasenetworks inplants. Arch Biochem Biophys.

[42] Taj G, Agarwal P, Grant M, Kumar A (2010). MAPK machinery in plants recognition and response to different stresses through multiple signal transduction pathways. Plant Signal Behav.

[43] Sreenivasulu N, Usadel B, Winter A, Radchuk V, Scholz U, Stein N, Weschke W, Strickert M, Close TJ, Stitt M, Graner A, Wobus U (2008). Barley grain maturation and germination: metabolic pathway and regulatory network commonalities and differences highlighted by new MapMan/PageMan profiling tools. Plant Physiol.

[44] Uchida M, Miyoshi T, Kaneniwa M, Ishihara K, Nakashimada Y, Urano N (2014). Production of 16.5% v/v ethanol from seagrass seeds. J Biosci Bioeng.

[45] Delefosse M, Povidisa K, Poncet D, Kristensen E, Olesen B (2016). Variation in size and chemical composition of seeds from the seagrass *Zostera marina*-ecological implications. Aquat Bot.

[46] Howell KA, Narsai R, Carroll A, Ivanova A, Lohse M, Usadel B, Millar AH, Whelan J (2009). Mapping metabolic and transcript temporal switches during germination in rice highlights specific transcription factors and the role of RNA instability in the germination process. Plant Physiol.

[47] Chen L, Wu JE, Li ZM, Liu Q, Zhao X, Yang HS (2019). Metabolomic analysis of energy regulated germination and sprouting of organic mung bean (*Vigna radiata*) using NMR spectroscopy. Food Chem.

[48] Glaubitz U, Li X, Schaedel S, Erban A, Sulpice R, Kopka J, Hincha DK, Zuther E (2017). Integrated analysis of rice transcriptomic and metabolomic responses to elevated night temperatures identifies sensitivity- and tolerance-related profiles. Plant Cell Environ.

[49] Yang PF, Li XJ, Wang XQ, Chen H, Chen F, Shen SH (2007). Proteomic analysis of rice (*Oryza sativa*) seeds during germination. Proteomics.

[50] Pujar A, Jaiswal P, Kellogg EA, Ilic K, Vincent L, Avraham S, Stevens P, Zapata F, Reiser L, Rhee SY, Sachs MM, Schaeffer M, Stein L, Ware D, McCouch S (2006). Whole-plant growth stage ontology for angiosperms and its application in plant biology. Plant Physiol.

[51] Barros M, Fleuri LF, Macedo GA (2010). Seed lipases: sources, applications and properties – a review. Braz J Chem Eng.

[52] Ghosh S, Pal A (2012). Identification of differential proteins of mungbean cotyledons during seed germination: a proteomic approach. Acta Physiol Plant.

[53] Erbas S, Tonguc M, Karakurt Y, Sanli A (2016). Mobilization of seed reserves during germination and early seedling growth of two sunflower cultivars. J Appl Bot Food Qual.

[54] Rosental L, Nonogaki H, Fait A (2014). Activation and regulation of primary metabolism during seed germination. Seed Sci Res.

[55] Gu JW, Chao HB, Gan L, Guo LX, Zhang K, Li YH, Wang H, Raboanatahiry N, Li MT (2016). Proteomic dissection of seed germination and seedling establishment in *Brassica napus*. Front Plant Sci.

[56] Han C, Yin XJ, He DL, Yang PF (2013). Analysis of proteome profile in germinating soybean seed, and its comparison with rice showing the styles of reserves mobilization in different crops. PLoS ONE.

[57] Finkelstein R, Reeves W, Ariizumi T, Steber C (2008). Molecular aspects of seed dormancy. Annu Rev Plant Biol.

[58] Chen K, Li GJ, Bressan RA, Song CP, Zhu JK, Zhao Y (2020). Abscisic acid dynamics, signaling, and functions in plants. J Integr Plant Biol.

[59] Tan BC, Joseph LM, Deng WT, Liu LJ, Li QB, Cline K, McCarty DR (2003). Molecular characterization of the *Arabidopsis* 9-cis epoxycarotenoid dioxygenase gene family. Plant J.

[60] Lopez-Molina L, Mongrand B, McLachlin DT, Chait BT, Chua NH (2002). ABI5 acts downstream of ABI3 to execute an ABA-dependent growth arrest during germination. Plant J.

[61] Finkelstein RR, Gampala SSL, Rock CD (2002). Abscisic acid signaling in seeds and seedlings. Plant Cell.

[62] Nakashima K, Yamaguchi-Shinozaki K (2013). ABA signaling in stress-response and seed development. Plant Cell Rep.

[63] Rubio S, Rodrigues A, Saez A, Dizon MB, Galle A, Kim TH, Santiago J, Flexas J, Schroeder JI, Rodriguez PL (2009). Triple loss of function of protein phosphatases type 2 C leads to partial constitutive response to endogenous abscisic acid. Plant Physiol.

[64] Ma Y (2009). Regulators of PP2C phosphatase activity function as abscisic acid sensors. Science.

[65] Jacobsen JV, Barrero JM, Hughes T, Julkowska M, Taylor JM, Xu Q, Gubler F (2013). Roles for blue light, jasmonate and nitric oxide in the regulation of dormancy and germination in wheat grain (*Triticum aestivum* L). Planta.

[66] Shu K, Liu XD, Xie Q, He ZH (2016). Two faces of one seed: hormonal regulation of dormancy and germination. Mol Plant.

[67] Bewley JD, Black M, Seeds (1994). Physiology of Development and Germination.

[68] Regnault T, Daviere JM, Heintz D, Lange T, Achard P (2014). The gibberellin biosynthetic genes *AtKAO1* and *AtKAO2* have overlapping roles throughout *Arabidopsis* development. Plant J.

[69] Chen Y, Hou MM, Liu LJ, Wu S, Shen Y, Ishiyama K, Kobayashi M, McCarty DR, Tan BC (2014). The Maize *DWARF1* encodes a gibberellin 3-Oxidase and is dual localized to the Nucleus and Cytosol(W). Plant Physiol.

[70] Rubinovich L, Weiss D (2010). The *Arabidopsis* cysteine-rich protein GASA4 promotes GA responses and exhibits redox activity in bacteria and in planta. Plant J.

[71] Chandrasekaran U, Zhao XT, Luo XF, Wei SW, Shu K (2022). Endosperm weakening: the gateway to a seed’s new life. Plant Physiol Biochem.

[72] Leubner-Metzger G (2002). Seed after-ripening and over-expression of class I beta-1,3-glucanase confer maternal effects on Tobacco testa rupture and dormancy release. Planta.

[73] Iglesias-Fernandez R, Rodriguez-Gacio MC, Barrero-Sicilia C, Carbonero P, Matilla A (2011). Three endo-beta-mannanase genes expressed in the micropylar endosperm and in the radicle influence germination of *Arabidopsis thaliana* seeds. Planta.

[74] Xu P, Cai XT, Wang Y, Xing L, Chen Q, Xiang CB (2014). HDG11 upregulates cell-wall-loosening protein genes to promote root elongation in *Arabidopsis*. J Exp Bot.

[75] Cosgrove DJ (2000). Loosening of plant cell walls by expansins. Nature.

[76] Muller K, Linkies A, Vreeburg RAM, Fry SC, Krieger-Liszkay A, Leubner-Metzger G (2009). In vivo cell wall loosening by hydroxyl radicals during cress seed germination and elongation growth. Plant Physiol.

[77] Chen F, Nonogaki H, Bradford KJ (2002). A gibberellin-regulated xyloglucan endotransglycosylase gene is expressed in the endosperm cap during tomato seed germination. J Exp Bot.

[78] Romo S, Jiménez T, Labrador E, Dopico B (2005). The gene for a xyloglucan endotransglucosylase/hydrolase from *Cicer arietinum* is strongly expressed in elongating tissues. Plant Physiol Biochem.

[79] Pergo EM, Ishii-Iwamoto EL (2011). Changes in energy metabolism and antioxidant defense systems during seed germination of the weed species *Ipomoea triloba* L. and the responses to allelochemicals. J Chem Ecol.

[80] Wang SQ, Wang B, Hua WP, Niu JF, Dang KK, Qiang Y, Wang ZZ (2017). De novo assembly and analysis of polygonatum sibiricum transcriptome and identification of genes involved in polysaccharide biosynthesis. Int J Mol Sci.

[81] Qin CX, Chen ZL, Wang M, Li AM, Liao F, Li YR, Wang MQ, Long MH, Lakshmanan P, Huang DL (2021). Identification of proteins and metabolic networks associated with sucrose accumulation in sugarcane (*Saccharum* spp. interspecific hybrids). J Plant Interact.

[82] Cheng XX, Xiong F, Wang CJ, Xie H, He S, Geng GH, Zhou Y (2018). Seed reserve utilization and hydrolytic enzyme activities in germinating seeds of sweet corn. Pak J Bot.

[83] Gallardo K, Job C, Groot SPC, Puype M, Demol H, Vandekerckhove J, Job D (2002). Importance of methionine biosynthesis for *Arabidopsis* seed germination and seedling growth. Physiol Plant.

[84] Galland M, Huguet R, Arc E, Cueff G, Job D, Rajjou L (2014). Dynamic proteomics emphasizes the importance of selective mRNA translation and protein turnover during *Arabidopsis* seed germination. Mol Cell Proteomics.

[85] Gupta A, Upadhyay RK, Prabhakar R, Tiwari N, Garg R, Sane VA, Sane AP (2022). SlDREB3, a negative regulator of ABA responses, controls seed germination, fruit size and the onset of ripening in tomato. Plant Sci.

[86] Mizoi J, Shinozaki K, Yamaguchi-Shinozaki K (2012). AP2/ERF family transcription factors in plant abiotic stress responses. Biochim Biophys Acta-Gene Regul Mech.

[87] Liu HZ, Wu W (2021). Comparative transcriptome analysis reveals function of TERF1 in promoting seed germination. Physiol Mol Biol Plants.

[88] Liu HZ, Yuan L, Guo W, Wu W (2022). Transcription factor TERF1 promotes seed germination under osmotic conditions by activating gibberellin acid signaling. Plant Sci.

[89] Xu S, Wang P, Zhou Y, Zhang X, Gu R, Liu X, Liu B, Song X, Xu S, Yue S. New insights into different reproductive effort and sexual recruitment contribution between two geographic *Zostera marina* L. populations in temperate China. Front Plant Sci. 2018; 9. 10.3389/fpls.2018.00015 PMC5816074 29483922

