# Supplementary material for: Combined transcriptome and proteome analysis reveal the key physiological processes in seed germination stimulated by decreased salinity in the seagrass Zostera marina L
Source: BMC Plant Biol. 2023 Nov 30;23:605. doi: 10.1186/s12870-023-04616-x (PMC10688091; doi:10.1186/s12870-023-04616-x)
Supplement: Supplementary file 1 — Supplementary Material: Table S1. Upregulated Differentially Expressed Proteins (DEPs) identified in main carbohydrate metabolism pathways in pre-germination stage (DoS vs. DeS). Table S2. Upregulated Differentially Expressed Proteins (DEPs) identified in main carbohydrate metabolism and lipid metabolism pathways in the germination stage (DeS vs. GeS). Table S3. Differentially Expressed Genes (DEGs) and Differentially Expressed Proteins (DEPs) identified in plant hormone signal transduction pathway (map04075) in the pre-germination stage. Table S4. Differentially Expressed Genes (DEGs) and Differentially Expressed Proteins (DEPs) identified in plant hormone signal transduction pathway (map04075) in the germination stage. Table S5. Primer sequences of RT-qPCR. [file 12870_2023_4616_MOESM1_ESM.docx]

**Supplementary materials for**

**C****ombined transcriptome and proteome analysis reveal the key physiological processes in seed germination stimulated by decreased salinity in the seagrass *Zostera marina* L.**

Yu Zhang^a,b,c,d,e,f^, Shidong Yue^a,b,c,d,e,f^, Mingjie Liu^a,b,c,d,e,f^, Xinhua Wang^a,b,c,d,e,f^, Shaochun Xu^a,b,c,d,e,f^, Xiaomei Zhang^a,b,c,d,e,f^, Yi Zhou^a,b,c,d,e,f^**^*^**

^a^ CAS Key Laboratory of Marine Ecology and Environmental Sciences, Institute of Oceanology, Chinese Academy of Sciences, Qingdao 266071, China

^b^ Laboratory for Marine Ecology and Environmental Science, Qingdao National Laboratory for Marine Science and Technology, Qingdao 266237, China

^c^ Center for Ocean Mega-Science, Chinese Academy of Sciences, Qingdao 266071, China

^d^ CAS Engineering Laboratory for Marine Ranching, Institute of Oceanology, Chinese Academy of Sciences, Qingdao 266071, China

^e^ University of Chinese Academy of Sciences, Beijing 100049, China

^f^ Shandong Province Key Laboratory of Experimental Marine Biology，Qingdao 266071, China

* Corresponding author. E-mail address: [yizhou@qdio.ac.cn](mailto:yizhou@qdio.ac.cn)

# Supplementary table legends

**Table S1**. Upregulated Differentially Expressed Proteins (DEPs) identified in main carbohydrate metabolism pathways in pre-germination stage (DoS vs. DeS)

**Table S2**. Upregulated Differentially Expressed Proteins (DEPs) identified in main carbohydrate metabolism and lipid metabolism pathways in the germination stage (DeS vs. GeS)

**Table S3.** Differentially Expressed Genes (DEGs) and Differentially Expressed Proteins (DEPs) identified in plant hormone signal transduction pathway (map04075) in the pre-germination stage

**Table S4.** Differentially Expressed Genes (DEGs) and Differentially Expressed Proteins (DEPs) identified in plant hormone signal transduction pathway (map04075) in the germination stage

**Table S5.** Primer sequences of RT-qPCR

**Table S1.** Upregulated Differentially Expressed Proteins (DEPs) identified in main carbohydrate metabolism pathways in pre-germination stage (DoS vs. DeS)

| Accession | Description | Log2FC(DeS/DoS) | P_value (DeS/DoS) | Regulate | Significant |
| --- | --- | --- | --- | --- | --- |
| **Starch and sucrose metabolism** | | | | | |
| Zosma06g17360 | Sucrose synthase | 2.39 | 1.31E-04 | up | yes |
| Zosma04g14260 | Fructokinase-2 | 1.17 | 5.17E-04 | up | yes |
| Zosma03g17270 | Alpha amylase-hypothetical protein | 0.97 | 7.31E-04 | up | yes |
| Zosma05g31400 | Starch synthase | 0.96 | 1.54E-04 | up | yes |
| Zosma04g08740 | Starch synthase | 0.94 | 4.76E-04 | up | yes |
| Zosma06g17190 | Starch synthase | 0.90 | 6.49E-04 | up | yes |
| Zosma03g27630 | Starch synthase | 0.78 | 1.87E-03 | up | yes |
| **Amino sugar and nucleotide sugar metabolism** | | | | | |
| Zosma03g22590 | UDP-N-acetylglucosamine pyrophosphorylase | 5.00 | 0.00E+00 | up | yes |
| Zosma05g02140 | UDP-D-glucuronate 4-epimerase 6 | 5.00 | 0.00E+00 | up | yes |
| Zosma01g24690 | dTDP-4-dehydrorhamnose reductase | 2.13 | 2.20E-03 | up | yes |
| Zosma04g14260 | Fructokinase-2 | 1.17 | 5.17E-04 | up | yes |
| Zosma06g10040 | UDP-glucose 6-dehydrogenase 3 | 0.73 | 2.04E-02 | up | yes |
| Zosma01g28230 | UDP-glucuronic acid decarboxylase 2 | 0.70 | 2.78E-03 | up | yes |
| Zosma01g01970 | UDP-glucose 6-dehydrogenase 3 | 0.66 | 6.00E-06 | up | yes |
| **Glycolysis/gluconeogenesis** | | | | | |
| Zosma06g24120 | Pyruvate kinase | 0.62 | 2.15E-02 | up | yes |
| Zosma01g11740 | Pyruvate kinase | 0.61 | 3.74E-02 | up | yes |
| **TCA cycle** | | | | | |
| Zosma01g42300 | Isocitrate dehydrogenase (NAD(+)) | 5.00 | 0.00E+00 | up | yes |

**Table S2.** Upregulated Differentially Expressed Proteins (DEPs) identified in main carbohydrate metabolism and lipid metabolism pathways in the germination stage (DeS vs. GeS)

| Accession | Description | Log2FC(DeS/DoS) | P_value(DeS/DoS) | Regulate | Significant |
| --- | --- | --- | --- | --- | --- |
| **Starch and sucrose metabolism** | | | | | |
| Zosma02g20080 | Sucrose synthase | 5.00 | 0.00E+00 | up | yes |
| Zosma02g17060 | endo-beta-1,4-glucanase, family GH9 | 0.62 | 3.01E-03 | up | yes |
| Zosma06g17360 | Sucrose synthase | 0.62 | 1.26E-02 | up | yes |
| **Amino sugar and nucleotide sugar metabolism** | | | | | |
| Zosma05g02140 | UDP-D-glucuronate 4-epimerase 6 | 1.64 | 7.28E-03 | up | yes |
| Zosma01g24690 | dTDP-4-dehydrorhamnose reductase | 0.80 | 3.98E-02 | up | yes |
| **Inositol phosphate metabolism** | | | | | |
| Zosma01g22790 | Inositol oxygenase | 5.00 | 0.00E+00 | up | yes |
| **alpha-Linolenic acid metabolism** | | | | | |
| Zosma01g01290 | Allene oxide synthase | 5.00 | 0.00E+00 | up | yes |

**Table S3.** Differentially Expressed Genes (DEGs) and Differentially Expressed Proteins (DEPs) identified in plant hormone signal transduction pathway (map04075) in the pre-germination stage

| **Gene name** | **Gene description** | **Log2FC(DeS/DoS)** | **Pvalue** | **Significant** | **Regulate** |
| --- | --- | --- | --- | --- | --- |
| **DEGs** | | | | | |
| Zosma02g24160 | Abscisic acid insensitive 5-like protein 2 | 3.70 | 3.30E-03 | yes | up |
| Zosma03g36780 | F-box protein GID2 (GID2, SLY1) | 2.48 | 4.14E-03 | yes | up |
| Zosma03g28540 | bZIP transcription factor | 2.08 | 3.38E-12 | yes | up |
| Zosma03g28550 | Transcription factor TGA5 | 2.03 | 3.53E-03 | yes | up |
| Zosma02g20780 | Auxin influx carrier (AUX1 LAX family) (AUX1, LAX) | 1.90 | 6.99E-13 | yes | up |
| Zosma01g05850 | Brassinosteroid insensitive 1 (BRI1) | 1.72 | 1.71E-18 | yes | up |
| Zosma01g07670 | EIN3-binding F-box protein (EBF1_2) | 1.66 | 1.26E-03 | yes | up |
| Zosma03g31320 | TIFY 3A-related | 1.43 | 5.98E-06 | yes | up |
| Zosma05g11640 | Serine/threonine-protein kinase SRK2 (SNRK2) | 1.33 | 2.08E-15 | yes | up |
| Zosma03g19400 | **Mitogen-activated protein kinase kinase 4/5, plant (MKK4_5P)** | 1.06 | 7.51E-06 | yes | up |
| Zosma01g05970 | Auxin responsive protein IAA15 | -1.08 | 7.38E-14 | yes | down |
| Zosma02g18130 | Abscisic acid insensitive 5-like protein 4 | -1.08 | 2.61E-20 | yes | down |
| Zosma04g24260 | ABA responsive element binding factor (ABF) | -1.09 | 6.18E-04 | yes | down |
| Zosma01g16020 | Transcription factor PIF1 | -1.11 | 9.93E-05 | yes | down |
| Zosma05g03420 | Abscisic acid insensitive 5 | -1.44 | 1.66E-29 | yes | down |
| Zosma02g24210 | Ethylene insensitive 3-like 1 protein related | -2.00 | 3.30E-09 | yes | down |
| Zosma01g09880 | Jasmonate ZIM domain-containing protein (JAZ) | -2.06 | 2.73E-76 | yes | down |
| Zosma05g30290 | SAUR family protein (SAUR) | -2.43 | 1.66E-13 | yes | down |
| Zosma06g08150 | Jasmonic acid-amino synthetase (JAR1) | -2.88 | 1.68E-02 | yes | down |
| Zosma02g01490 | Jasmonate ZIM domain-containing protein (JAZ) | -5.87 | 4.45E-03 | yes | down |
| Zosma05g22130 | Indole 3 acetic acid amido synthetase GH3.2 related | -7.33 | 5.81E-09 | yes | down |
| **Proteins** | | | | | |
| Zosma03g19400 | **Mitogen-activated protein kinase kinase** | 0.85 | 3.88E-02 | yes | up |
| Zosma04g23150 | Kinase family protein | 0.24 | 5.41E-01 | no | no change |
| Zosma03g20260 | Histidine-containing phosphotransfer protein 5 | 0.24 | 1.75E-01 | no | no change |
| Zosma06g23000 | Histidine kinase 2 | 0.15 | 3.73E-01 | no | no change |
| Zosma06g08390 | Mitogen-activated protein kinase 1 | 0.07 | 4.04E-01 | no | no change |
| Zosma06g29160 | Kinase family protein | 0.02 | 8.91E-01 | no | no change |
| Zosma05g31900 | putative serine/threonine-protein kinase | -0.16 | 4.72E-01 | no | no change |

**Table S4.** Differentially Expressed Genes (DEGs) and Differentially Expressed Proteins (DEPs) identified in plant hormone signal transduction pathway (map04075) in the germination stage

| **Gene name** | **Gene description** | **Log2FC(GeS/DeS)** | **Pvalue** | **Significant** | **Regulate** |
| --- | --- | --- | --- | --- | --- |
| **DEGs** | | | | | |
| Zosma05g18000 | Brassinosteroid resistant 1/2 (BZR1_2) | 14.24 | 4.99E-42 | yes | up |
| Zosma02g01490 | Jasmonate ZIM domain-containing protein (JAZ) | 7.70 | 6.29E-09 | yes | up |
| Zosma05g22130 | Indole 3 acetic acid amido synthetase GH3.2 related | 5.75 | 2.53E-15 | yes | up |
| Zosma05g10360 | Transcription factor MYC3-related | 5.38 | 5.98E-04 | yes | up |
| Zosma01g31340 | SAUR family protein (SAUR) | 5.34 | 1.78E-33 | yes | up |
| Zosma01g36540 | Protein, putative-related | 4.97 | 1.31E-02 | yes | up |
| Zosma04g15520 | Auxin influx carrier (AUX1 LAX family) (AUX1, LAX) | 4.49 | 9.07E-14 | yes | up |
| Zosma06g08150 | Jasmonic acid-amino synthetase (JAR1) | 4.20 | 5.12E-10 | yes | up |
| Zosma05g30290 | SAUR family protein (SAUR) | 3.75 | 1.44E-45 | yes | up |
| Zosma01g06360 | Jasmonic acid-amino synthetase (JAR1) | 3.63 | 1.18E-07 | yes | up |
| Zosma01g16060 | Protein phosphatase 2C (PP2C) | 2.97 | 1.84E-26 | yes | up |
| Zosma06g09020 | Protein phosphatase 2C16-RELATED | 2.70 | 2.19E-74 | yes | up |
| Zosma03g16370 | SAUR family protein (SAUR) | 2.67 | 1.20E-04 | yes | up |
| Zosma02g03900 | Histidine kinase 3 | 2.10 | 5.34E-70 | yes | up |
| Zosma03g24130 | Regulatory protein NPR3-related | 2.09 | 4.87E-53 | yes | up |
| Zosma06g24030 | Auxin-responsive protein IAA (IAA) | 1.75 | 1.87E-12 | yes | up |
| Zosma03g14830 | Phytochrome-interacting factor 3 (PIF3) | 1.38 | 2.77E-04 | yes | up |
| Zosma06g01320 | Abscisic acid receptor PYL1-related | 1.37 | 1.98E-06 | yes | up |
| Zosma06g09200 | Two-component response regulator ARR 14 | 1.19 | 4.04E-19 | yes | up |
| Zosma02g17740 | Two-component response regulator ARR18 | 1.15 | 8.61E-23 | yes | up |
| Zosma03g19400 | Mitogen-activated protein kinase kinase 4/5, plant (MKK4_5P) | 1.04 | 8.35E-13 | yes | up |
| Zosma04g24260 | ABA responsive element binding factor (ABF) | -1.29 | 3.95E-04 | yes | down |
| Zosma03g20240 | Gibberellin receptor GID1 (GID1) | -1.34 | 1.21E-30 | yes | down |
| Zosma01g16020 | Transcription factor PIF1 | -1.65 | 5.25E-07 | yes | down |
| Zosma04g25480 | Abscisic acid receptor PYR/PYL family (PYL) | -1.79 | 2.42E-23 | yes | down |
| Zosma06g06380 | Helix-loop-helix DNA-binding domain (HLH) | -2.18 | 1.41E-03 | yes | down |
| Zosma06g29330 | SAUR family protein (SAUR) | -3.61 | 1.51E-17 | yes | down |
| Zosma02g24210 | Ethylene insensitive 3-like 1 protein related | -4.70 | 1.04E-02 | yes | down |
| **Protein** | | | | | |
| Zosma05g31900 | putative serine/threonine-protein kinase | 0.16 | 4.68E-01 | no | no change |
| Zosma06g23000 | Histidine kinase 2 | -0.45 | 8.76E-03 | yes | no change |
| Zosma06g08390 | Mitogen-activated protein kinase 1 | 0.07 | 3.44E-01 | no | no change |
| Zosma06g29160 | Kinase family protein | 0.18 | 3.27E-01 | no | no change |
| Zosma03g20260 | Histidine-containing phosphotransfer protein 5 | -0.16 | 3.42E-01 | no | no change |
| Zosma03g19400 | Mitogen-activated protein kinase kinase | 0.23 | 2.01E-01 | no | no change |
| Zosma04g23150 | Kinase family protein | -0.06 | 8.10E-01 | no | no change |

**Table S5.** Primer sequences of RT-qPCR

| Gene name | Primer sequence (5'——3') | Product length (bp) |
| --- | --- | --- |
| Zosma01g35220 | F:GTTAGAGTGGTGGATGGGAGT | 226 |
|  | R:GAGACGGAGCCAAGGTTT |  |
| Zosma05g17980 | F:CTACGCAACAACGCTGAC | 296 |
|  | R:CCAATCCCACAATTACCA |  |
| Zosma03g22750 | F:AAAATCAGGATTGGCTCG | 228 |
|  | R:ATGGCTGTTTCTAATTCATCTC |  |
| Zosma02g23380 | F:GACGAAATTCCCAAGCAC | 135 |
|  | R:ATCGGAGATGGAGTCACAA |  |
| Zosma03g03930 | F:GACTTCACTCCTCCTCTACC | 200 |
|  | R:GTTCGTCTATGACTGTGCC |  |
| Zosma03g25310 | F:ACCCAAACTATCCAGACAT | 206 |
|  | R:GCCACGACGAGTGACAAC |  |
| Zosma03g22940 | F:TTGGGATGATGTAGTGGC | 215 |
|  | R:TGTTGCTTCCTGGATTGA |  |
| Zosma03g18590 | F:AGGTACGGAAGCGAGTGA | 194 |
|  | R:GTGAACCGAGGAATGGAG |  |
| Reference gene: 18S rRNA | F:CAACCATAAACGATGCCGA | 100 |
|  | R:AGCCTTGCGACCATACTCC |  |
